# Cell size explains shift in phytoplankton community structure following storm‐induced changes in light and nutrients

**DOI:** 10.1002/ecy.70043

**Published:** 2025-03-11

**Authors:** Alexis L. N. Guislain, Jens C. Nejstgaard, Jan Köhler, Erik Sperfeld, Ute Mischke, Birger Skjelbred, Hans‐Peter Grossart, Anne Lyche Solheim, Mark O. Gessner, Stella A. Berger

**Affiliations:** ^1^ Department of Plankton and Microbial Ecology Leibniz Institute of Freshwater Ecology and Inland Fisheries (IGB) Stechlin Germany; ^2^ Berlin‐Brandenburg Institute of Advanced Biodiversity Research (BBIB) Berlin Germany; ^3^ Department of Community and Ecosystems Ecology Leibniz Institute of Freshwater Ecology and Inland Fisheries (IGB) Berlin Germany; ^4^ Animal Ecology Zoological Institute and Museum, University of Greifswald Greifswald Germany; ^5^ Department of Ecohydrology and Biogeochemistry Leibniz Institute of Freshwater Ecology and Inland Fisheries (IGB) Berlin Germany; ^6^ Norwegian Institute for Water Research (NIVA) Oslo Norway; ^7^ Department of Biochemistry and Biology Potsdam University Potsdam Germany; ^8^ Department of Ecology Berlin Institute of Technology (TU Berlin) Berlin Germany; ^9^ Present address: Section IV 2.5 Trace Analysis, Artificial Ponds and Streams, German Environment Agency (UBA) Berlin Germany; ^10^ Present address: Bavarian Environment Agency, Ecology of Lakes and Rivers Wielenbach Germany

**Keywords:** browning, cell‐size distribution, enclosure experiment, extreme weather events, nutrient gradient, paradox of the plankton, species coexistence, trait‐based ecology

## Abstract

Understanding the mechanisms driving community structure and dynamics is crucial in the face of escalating climate change, including increasing incidences of extreme weather. Cell size is a master trait of small organisms that is subject to a trade‐off between resistance to grazing and competition for resources, and thus holds potential to explain and predict community dynamics in response to disturbances. Here, we aimed at determining whether cell size can explain shifts in phytoplankton communities following changes in nutrient and light conditions resulting from storm‐induced inputs of nutrients and colored dissolved organic matter (cDOM) to deep clearwater lakes. To ensure realistic environmental conditions, we used a crossed gradient design to conduct a large‐scale enclosure experiment over 6 weeks. Cell size explained phytoplankton community structure when light availability declined as a result of cDOM supply. Initially unimodal, with small‐celled species accounting for up to 60% of the total community biovolume, the cell‐size distribution gradually shifted toward large‐celled species as light levels declined following cDOM addition. Neither nutrients nor mesozooplankton affected the shift in cell‐size distribution. These results suggest a distinct competitive advantage of larger over smaller species at reduced light levels following cDOM inputs during storm events. Importantly, the clustering of species in two distinct size classes implies that interspecific size differences matter as much as cell size per se to understand community dynamics. Given that shifts in cell‐size distribution have strong implications for food‐web structure and biogeochemical cycles, our results point to the importance of analyzing cell‐size distributions of small organisms as an essential element to forecast community and ecosystem dynamics in response to environmental change.

## INTRODUCTION

Since the formulation of the competitive exclusion principle by Gause (1934), ecologists have been puzzled by the enormous diversity of species on Earth. This led Hutchinson (1961) to articulate his famous “paradox of the plankton” relating to the striking discrepancy between the species richness of communities and the apparently limited number of resources for which species compete. Originally focused on plankton, Hutchinson's paradox has been expanded to the diversity of organisms ranging from bacteria to birds (May, 1988; Mora et al., 2011; Ptacnik et al., 2010). Despite a wealth of theoretical and empirical work using a variety of communities to resolve this conundrum, ecologists remain challenged in the endeavor to unravel the drivers of community structure (e.g., Chesson, 2000; Hubbell, 2001; MacArthur & Levins, 1967; Scheffer et al., 2018), which continues to spark fundamental interest in understanding the dynamics and underlying mechanisms. In addition, it has become particularly pressing to determine these relationships in view of the current global biodiversity and climate crisis. This includes responses to extreme weather events, which are increasing in magnitude and frequency and can have far‐reaching consequences for biodiversity and the functioning of ecosystems (IPCC, 2023).

A type of weather event that lends itself to experimental manipulation to assess the role of extreme events in determining community structure is severe storms affecting lakes (Stockwell et al., 2020). Apart from physical effects (Jennings et al., 2012; Kasprzak et al., 2017), storms are often accompanied by heavy rainfall and terrestrial runoff that carries nutrients and colored dissolved organic matter (cDOM), thereby altering resource supply in receiving ecosystems (Cooney et al., 2018; Drakare et al., 2002; Solomon et al., 2015). By strongly absorbing light, especially in the blue range of the spectrum, cDOM rapidly attenuates photosynthetically active radiation (PAR) (Kirk, 1994; Thrane et al., 2014). Increases in cDOM concentrations thus not only limit photosynthesis and primary production (Kritzberg et al., 2014; Thrane et al., 2014), but also alter competitive interactions among species that otherwise coexist by niche partitioning along the PAR spectrum (Burson et al., 2019; Luimstra et al., 2020; Stomp et al., 2004).

Nutrient enrichment following storm events may also alter dominance patterns of species and increase biomass, shifting the drivers of community structure from nutrient to light competition (Brauer et al., 2012; Hautier et al., 2009). In addition, storm‐induced runoff can alter producer and consumer dynamics in contrasting ways (Solomon et al., 2015). On the one hand, light attenuation by cDOM supplied to lakes may reduce food availability for herbivores and, by limiting grazer abundances, release feeding pressure on primary producers (Kelly et al., 2014). On the other hand, lowered light‐to‐nutrient supply ratios resulting in lower carbon‐to‐phosphorus ratios of producers (Sterner et al., 1997) and reduced UV radiation can benefit herbivores by improving food quality (Minguez et al., 2020) or reducing cell damage (Wolf & Heuschele, 2018).

Much effort has been invested in trait‐based approaches to gain insight into community structure and responses to global change (McGill et al., 2006; Westoby & Wright, 2006), including for phytoplankton in lakes and oceans (Litchman, 2022; Litchman & Klausmeier, 2008). Traits capture fundamental biological functions (Litchman & Klausmeier, 2008) and thus are often instrumental for community dynamics driven by resource competition (Edwards et al., 2013a, 2013b; Schwaderer et al., 2011). Assessing the effects of storm‐induced runoff on community structure and dynamics requires considering not only changes in competitive relationships but also in trophic interactions. Cell size has been identified as a master trait governing resource acquisition, growth, reproduction, and trophic interactions (Berger et al., 2014; Hillebrand et al., 2022; Litchman & Klausmeier, 2008; Marañón, 2015). In particular, cell size is often subject to a trade‐off between competitive ability to acquire resources and resistance to grazing. Small‐celled species are expected to be favored when light and nutrient availability are low because they often have lower half‐saturation constants for nutrient uptake (Edwards et al., 2012) and steeper initial slopes of photosynthesis‐irradiance curves (Schwaderer et al., 2011) than larger species. In contrast, when resources are abundant, larger species could be relatively more abundant in communities if size provides sufficient protection from grazing mortality compared with smaller cells (Pančić & Kiørboe, 2018). However, these expectations are at variance with several observations in situ (see Marañón, 2015 for a review). For example, grazing mortality even of large cells can be important (e.g., Chang et al., 2013). Furthermore, size scaling of growth rate has been suggested to favor the dominance of intermediate cell sizes during phytoplankton blooms, resulting in a unimodal cell‐size distribution (Marañón, 2015; Marañón et al., 2013).

In this study, we asked whether cell size can explain shifts in phytoplankton community structure in the wake of severe storms that reshape environmental conditions. Our first objective was to determine how species richness responds to concomitant inputs of nutrients and cDOM, the expectation being that nutrient effects are dampened by cDOM‐mediated light attenuation. Specifically, we tested whether species numbers decline as storm‐induced nutrient and cDOM supplies from the catchment increase. The second aim was to determine whether distinct shifts in cell size are a pattern underlying changes in community structure and dynamics after storm events. Specifically, we tested whether small‐celled species dominate over larger‐celled species as cDOM‐induced light limitation increases and whether large‐celled species dominate over smaller‐celled species at elevated nutrient and PAR availability. To address these aims, we performed a lake enclosure experiment with a natural plankton community, where we mimicked different levels of cDOM and nutrient supply, reflecting inputs to lakes by terrestrial runoff during storm events. We hypothesized that (1) phytoplankton species numbers will decrease with increasing cDOM supply and nutrient availability because of light limitation of photosynthesis and restricted light‐dependent nutrient uptake; (2) the size distribution of species under conditions of low light and high nutrient availability will shift toward small cells, which tend to have lower minimum light requirements than larger cells; and (3), conversely, large cells will increase in abundance at elevated light and nutrient levels because of sufficient access to both resources and a lower grazing mortality than experienced by small cells.

## METHODS

### Experimental setup

The experiment was performed in a large enclosure facility located in Lake Stechlin, a deep clearwater lake in northeastern Germany (53°08′36′′ N, 13°01′41′′ E). The facility is composed of 24 enclosures, each 9 m in diameter and about 20‐m deep, thereby enclosing nearly 1300 m^3^ of water (Lyche Solheim et al., 2024). Before the start of the experiment, any fish trapped in the enclosures were removed with gill nets (6.5‐mm mesh size) and water in the enclosures was exchanged with epilimnetic and hypolimnetic water of the surrounding lake to ensure similar initial conditions among all experimental units. Water pumped into the enclosures was passed through a 3‐mm mesh to prevent the reintroduction of fish, including larvae.

We created an experimental gradient of seven nutrient levels at each of three levels of browning (none, intermediate, high) by adding a single pulse of nutrients and cDOM to a total of 21 enclosures. Browning was achieved by adding industrially processed humic substances marketed as HuminFeed (HF, HuminTech GmbH, Grevenbroich, Germany; Scharnweber et al., 2021). HF is characterized by high solubility in water, efficient absorbance of PAR, recalcitrance of the carbon, and low nutrient concentrations. We obtained three levels of browning by adding 0 (no HF addition), 5, and 10 mg HF L^−1^ to the enclosures, referred to as A, B, and C, respectively. The mean (±SD) dissolved organic carbon (DOC) concentrations throughout the experiment were slightly higher in the B (5.7 ± 0.5 mg L^−1^) and C (6.1 ± 0.6 mg L^−1^) than in the A enclosures (5.3 ± 0.5 mg L^−1^). H_3_PO_4_ was added to increase the initial P concentration of 18 μg L^−1^ by 0, 1, 4, 9, 16, 25, and 36 μg P L^−1^, respectively. Nitrogen was added as NH_4_NO_3_ in amounts respecting the lake's molar ratio of bioavailable N:P, which was close to the Redfield ratio of 16:1. The nutrient and browning pulse was completed between 8 and 10 June 2015.

### Abiotic and biotic variables

The 21 enclosures used in the experiment were equipped with temperature (YSI Inc., Yellow Springs, OH, USA) and PAR (LI‐COR Inc., Lincoln, NE, USA) sensors mounted on profilers recording hourly vertical profiles at 0.5‐m depth steps (Giling et al., 2017). The light attenuation coefficient was estimated as the slope of the linear regression between ln(PAR) and depth. Depth‐integrated water samples from the epilimnion (6–7 m) were collected by hose samplers once a week from 5 June until 21 July 2015. Concentrations of total phosphorus (TP), soluble reactive phosphorus (SRP), total nitrogen (TN), ammonium, nitrite, and nitrate were determined by flow injection analysis following ISO procedures (FIAStar 5000, FOSS, Höganäs, Sweden).

We tested the association between mean epilimnion PAR during daytime and SRP in all enclosures and throughout the experiment by determining the log–log relationship between SRP and PAR using linear mixed‐effects (LMEs) models that account for the enclosure dependence of measurements. We also used LME models to quantify the linear increase in epilimnetic temperature over time to account for our repeated measurements. We determined the effect of browning on this relationship by comparing slopes of the regression lines by analyses of covariance with the Kenward‐Rogers method. All LME models were computed using the R package *lme4* (Bates et al., 2015).

We analyzed phytoplankton species composition and biovolume from epilimnetic water samples (250 mL) taken weekly from 5 June to 21 July and fixed with acidic Lugol's solution. Species were identified and enumerated under an inverted microscope (Nikon Diaphot, Tokyo, Japan, and Leica DMI3000 B, Wetzlar, Germany). Species‐specific cell volumes (referred to as cell size below) were estimated from approximations of geometric shapes (European Committee for Standardization, 2015). Species‐specific biovolumes were then determined as the product of species‐specific cell volume and cell abundance. Mesozooplankton were sampled by vertical net tows (90‐μm mesh size) from 1.5 m above the sediment to the water surface and preserved in sugar‐formalin solution (final concentrations of 50% and 4%, respectively). Biomass of the mesozooplankton species was determined by identifying, counting, and sizing specimens under an inverted microscope. Biomass was calculated based on length measurements of 10–30 specimens per taxon and length‐dry mass relationships established for Lake Stechlin and other populations (Bottrell et al., 1976; Kasprzak, 1983). Biomass carbon was assumed to be 50% of dry mass (Winberg, 1971).

The effects of browning (across the seven levels of P supply) and P enrichment (across the three levels of browning) on the log‐transformed mesozooplankton biomass were determined in two separate LME models accounting for repeated measurements. Each browning or nutrient enrichment level was compared with the model's intercept, reflecting the reference condition with no HF or P addition, respectively. We adopted the same approach for the log‐transformed total biovolume of phytoplankton.

### Cell‐size distribution

We tested for each enclosure whether the phytoplankton cell‐size distribution deviated significantly from a unimodal distribution. At the start and end of the experiment, we investigated the size distribution of species biovolumes using generalized additive models (GAMs) (Wood, 2017). We determined for each enclosure the relative biovolume of species categorized by size intervals of 1 log_2_ μm^3^ over the range of 1–15 log_2_ μm^3^. We normalized the relative biovolumes of species within each enclosure (i.e., proportion of the total biovolume), which ranged from 1.1 × 10^−5^ to 0.76, by applying a logit transformation. Since biovolumes were found to vary significantly across the light but not the nutrient gradient, we fitted one GAM per browning level at both the start and end of the experiment, using the relative biovolume of species across all seven enclosures regardless of the P enrichment level. P level was set as a random effect to account for any nutrient‐dependent variation in cell size distribution. All GAM diagnostic plots (function *appraise* in the *gratia* R package; Simpson, 2023) indicated satisfactory agreement with the normal distribution of deviance residuals, homogeneity of deviance residuals along the linear predictor, and linearity of observed versus fitted values.

To test for the number of modes along the cell‐size gradient, we computed the first‐order derivatives of the GAMs. A significant peak in a GAM model translates to a significant positive derivative along the ascending part of the peak, followed by a negative derivative along the descending part. Since including the random effect for nutrients never improved the GAMs according to the Akaike information criterion (AIC), we averaged the seven derivatives per browning level and computed 95% GAM confidence envelopes. All GAM analyses were performed with the *mgcv* package in R (Wood, 2017).

### Species richness

We explored variation in phytoplankton species numbers in response to PAR, SRP, and mesozooplankton biomass using an LME model. Despite a significant negative correlation between PAR and SRP, we included the interaction between PAR and SRP in the model. Elapsed time was included as a fixed predictor because species numbers gradually declined during the experiment. All predictors were standardized by centering them around their mean and dividing by their SD (*z*‐score). The model included random intercepts for enclosures to account for repeated measurements. To test whether elapsed time had a significant effect, we computed a likelihood ratio test (LRT) comparing the outcome of models that included or excluded elapsed time as a variable. We also tested for the effect of mesozooplankton biomass using the same method.

To assess variation in the size distribution of phytoplankton, we explored in a presence–absence (PA) model whether the cell size of species had an effect on the probability that a given species occurred at any time during the experiment and along the PAR, SRP, and mesozooplankton biomass gradients. Specifically, we tested whether at lower light and nutrient levels the probability of a species' occurrence was higher for small‐celled than larger species and whether the occurrence of large‐celled species was associated with a putatively higher grazing pressure on small‐celled species. Species were separated into two size ranges for this analysis, one comprising small‐celled species (0–8 log_2_ μm^3^) and one comprising species with moderate cell size (8–15 log_2_ μm^3^).

We built a generalized linear mixed model (GLMM) using the R package *lme4* (Bates et al., 2015). We included random intercepts for species identity to capture variation in species occurrences. Since species PA can be described by a binomial distribution with values 0 (absence) and 1 (presence), the PA model was

(1) $$\text{logit}\left({\mathrm{PA}}_{i}^{j}\right)=\beta_{0}^{j}+\beta_{\text{size}}{\text{size}}_{i}+\beta_{\mathrm{PAR}}{\mathrm{PAR}}_{i}+\beta_{\mathrm{day}}{\mathrm{day}}_{i}+\beta_{\mathrm{zoo}}{\mathrm{zoo}}_{i}+\beta_{\text{size}\times \mathrm{PAR}}{\text{size}}_{i}\times {\mathrm{PAR}}_{i}+\beta_{\text{size}\times \mathrm{day}}{\text{size}}_{i}\times {\mathrm{day}}_{i}+\beta_{\text{size}\times \mathrm{zoo}}{\text{size}}_{i}\times {\mathrm{zoo}}_{i}+\varepsilon_{i}^{j},$$

where $\text{logit}\left({\mathrm{PA}}_{i}^{j}\right)$ is the logit link function of the expected probability of occurrence of species *j* for observation *i* $\left({\mathrm{PA}}_{i}^{j}\right)$, $\beta_{0}^{j}$ is the species‐specific intercept for species *j*, β_size_ the difference in logit(PA) between the two size ranges when all continuous predictors are zero, size_*i*_ the dummy variable for the cell‐size range of small (0–8 log_2_ μm^3^) or large (8–15 log_2_ μm^3^) species, PAR_*i*_ the mean PAR in the epilimnion during daytime, day_*i*_ the day of experiment, zoo_*i*_ the mesozooplankton biomass for observation *i* (all three continuous predictors were standardized), β_PAR_ the coefficient for PAR, β_day_ the coefficient for day, β_zoo_ the coefficient for mesozooplankton biomass, β_size×PAR_, β_size×day_ and β_size×zoo_ the coefficients for size×PAR, size×day and size×zoo, respectively, and $\varepsilon_{i}^{j}$ the residual logit(PA). The species‐specific intercept for species *j*, $\beta_{0}^{j}$, is expressed as $\beta_{0}^{j}=\beta_{0}+u_{0}^{j}$. Term $u_{0}^{j}$ is the random effect for species *j*, which is normally distributed with mean zero and variance σ_0_^2^ and estimates the unexplained interspecific variation in the intercept. Term β_0_ is the fixed intercept. Predictors were standardized (*z*‐score) to facilitate the interpretation of results and limit the influence of extreme values. Only species with at least one occurrence per sampling day (out of 21 possible ones when present in all enclosures) were included in the analysis (189 species) to limit the influence of extremely rare species on the model results.

### Biovolume models

We also ran biovolume models contingent on species occurrence to assess the effect of cell size on the biovolume of species over time and along separate gradients of PAR, SRP, and zooplankton biomass. This two‐part modeling approach (Cunningham & Lindenmayer, 2005; Edwards et al., 2013a) enabled us to deal with the semicontinuous distribution of biovolume data containing many zeroes and to detect potential differences between predictors of species occurrence and biovolume (Mutshinda et al., 2022). We tested whether the relative biovolume of species with smaller cells increased at lower light and nutrient levels and whether larger species benefited from higher grazing pressure on smaller‐celled species. We investigated the cell‐size dependence of species‐specific relative biovolumes along the standardized predictors of PAR, zooplankton biomass, SRP, and elapsed time by computing four separate biovolume models, all excluding species with zero biovolumes. Only species observed at least three times during the experiment were included (120 out of 189 species). Interpretation of the model outcomes proved robust to this threshold; changing the minimum number of species occurrences between 3 and 9 did not notably affect the results. Relative biovolumes were logit‐transformed to ensure a normal distribution of the data (Warton & Hui, 2011).

To avoid the need to fit a total of 120 regressions, one for each species, random intercepts for species and random slopes for predictors (PAR, zooplankton, SRP or time) were included in an LME model, thus accounting for differences in both (1) biovolume at mean predictor values across species (random intercepts) and (2) slopes of the relationships with the predictors across species (random slopes). The biovolume model for PAR was

(2) $$\text{logit}\left({\text{relBV}}_{i}^{j}\right)=\beta_{0}^{j}+\beta_{\text{size}}{\text{size}}_{i}+\beta_{\mathrm{PAR}}^{j}{\mathrm{PAR}}_{i}+\beta_{\text{size}\times \mathrm{PAR}}{\text{size}}_{i}\times {\mathrm{PAR}}_{i}+\varepsilon_{i}^{j},$$

where $\text{logit}\left({\text{relBV}}_{i}^{j}\right)$ is the logit relative biovolume of species *j* for observation *i*, $\beta_{0}^{j}$ is the species‐specific intercept for species *j*, β_size_ the coefficient for size, size_*i*_ the standardized log_2_ cell size for observation *i*, $\beta_{\mathrm{PAR}}^{j}$ the coefficient for species *j* against PAR, PAR_*i*_ the standardized mean PAR in the epilimnion during daytime for observation *i*, β_size×PAR_ the coefficient for size×PAR, and $\varepsilon_{i}^{j}$ the residual logit(relBV). The terms $\beta_{0}^{j}$ and $\beta_{\mathrm{PAR}}^{j}$ are expressed as $\beta_{0}^{j}=\beta_{0}+u_{0}^{j}$ and $\beta_{\mathrm{PAR}}^{j}=\beta_{\mathrm{PAR}}+u_{\mathrm{PAR}}^{j}$, where the random effects $u_{0}^{j}$ and $u_{\mathrm{PAR}}^{j}$ are normally distributed with mean zero and variance σ_0_^2^ and σ_PAR_^2^, respectively. These random effects estimate unexplained interspecific variation in the intercept and slope of the relationship with PAR, respectively. Term β_0_ is the fixed intercept of logit(relBV) when all predictors are zero, and β_PAR_ is the average coefficient for PAR across all species. The biovolume models for mesozooplankton biomass, SRP and elapsed time had the same model structure. The autocorrelation function (ACF) plots of the residuals showed no significant autocorrelation. The diagnostic plots (function check_model in the *performance* R package; Lüdecke et al., 2021) for all LME models showed satisfactory agreement with the assumptions of linearity, homogeneity of variance, normality of residuals and normality of random effects.

As with the PA models, we used LRTs to test whether phytoplankton responses to the three predictors (PAR, SRP, zooplankton) were species‐specific by comparing the outcomes of the biovolume models with the corresponding alternative models that did not include random slopes. We then tested whether cell size had an effect on the relative biovolume of species along the predictors by computing LRTs comparing the biovolume models and the corresponding alternative models that did not include the interaction between cell size and each of the predictors. The LRT results were all supported by parametric bootstrap tests with 999 simulations.

All analyses were performed using R version 4.2.2 (R Core Team, 2022).

## RESULTS

### Environmental conditions

**FIGURE 1:**
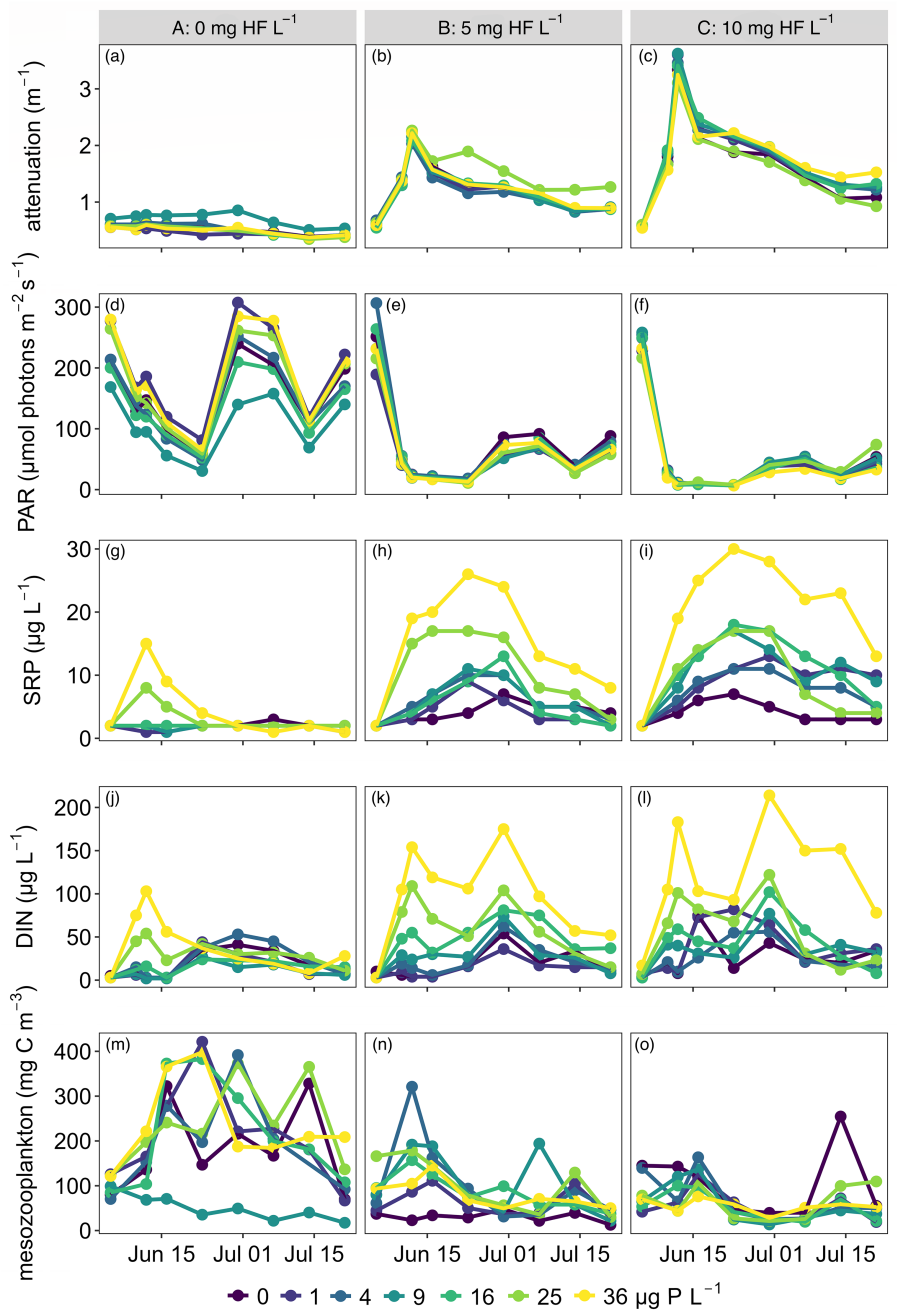
Temporal dynamics of the light attenuation coefficient (a–c) and mean photosynthetically active radiation (PAR) during daytime (d–f), soluble reactive phosphorus (SRP) (g–i), dissolved inorganic nitrogen (DIN) (j–l), and mesozooplankton biomass (m–o) in enclosures receiving no (A: 0 mg HF L^−1^; left column: a, d, g, j, m), intermediate (B: 5 mg HF L^−1^; middle column: b, e, h, k, n), or high (C: 10 mg HF L^−1^; right column: c, f, i, l, o) levels of colored dissolved organic matter in the form of HuminFeed (HF). The experimental P enrichment (addition of 0, 1, 4, 9, 16, 25, and 36 μg P L^−1^) is represented by the color gradient from dark blue to yellow.

The addition of cDOM in the form of HF increased the light attenuation in the enclosures receiving cDOM (B and C) as compared with the clear A enclosures (Figure 1a–c). As a result, the exponential decrease in PAR with depth was accentuated, thus reducing the average PAR level in the epilimnion (Figure 1d–f). Over time, however, light attenuation decreased in the enclosures receiving cDOM (B and C), whether due to photobleaching of cDOM or other mechanisms such as flocculation (Fonvielle et al., 2021). Mean PAR and SRP levels in the epilimnion were negatively correlated (Figure 1d–i), consistent with our hypothesis (H1) that light limitation following cDOM addition reduced the uptake of nutrients.

The log–log relationship between PAR and SRP indicated that SRP declined significantly with increasing PAR (LME, slope ± SE (95% CI) = −0.26 ± 0.07 (−0.39; −0.13), *p* < 0.001). Temperature significantly increased over time in all enclosures (LME, *p* < 0.001; Appendix S1: Figure S1) from 15.6 ± 0.1°C on 5 June up to 20.6 ± 0.1°C on 21 July (mean ± SD across all enclosures). This increase did not significantly differ among enclosure types (*p* > 0.90; Appendix S1: Tables S1 and S2).

### Plankton biomass

Mesozooplankton biomass rapidly increased after the start of the experiment (Figure 1m–o) but significantly less so in the B (LME, *p* < 0.001) and C (LME, *p* < 0.001) enclosures than in the A enclosures (Appendix S1: Figure S2a). Phytoplankton biovolume declined in the enclosures receiving cDOM, where light availability was reduced (LME, *p* < 0.001; Appendix S1: Figure S3a,b). Nutrient enrichment had no effect on zooplankton biomass (LME, all *p* > 0.50; Appendix S1: Figure S2b) or phytoplankton biovolume (LME, all *p* > 0.40; Appendix S1: Figure S3c). The dynamics in one of the clear enclosures (A4) were distinctly different in that zooplankton biomass was markedly lower than in the other clear enclosures. This was due to the dominance of a large predatory calanoid copepod (*Heterocope appendiculata*) that reduced the abundance of herbivorous zooplankton species and thus overall mesozooplankton biomass.

### Phytoplankton size distribution

**FIGURE 2:**
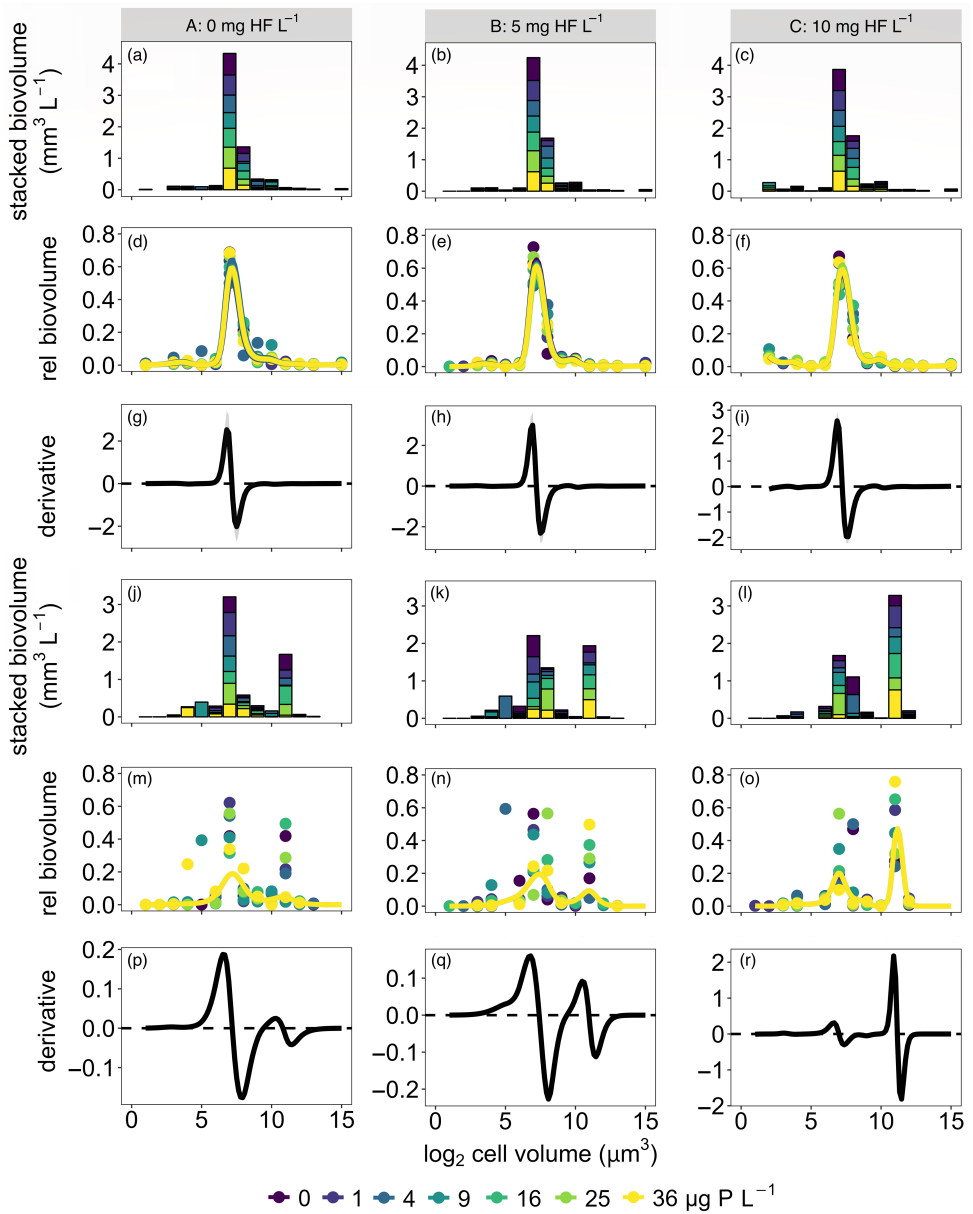
Cell‐size distribution of the relative biovolume of phytoplankton species in enclosures receiving no (A: 0 mg HF L^−1^; left column: a, d, g, j, m, p), intermediate (B: 5 mg HF L^−1^; middle column: b, e, h, k, n, q), or high (C: 10 mg HF L^−1^; right column: c, f, i, l, o, r) levels of colored dissolved organic matter before the simulated storm event on 5 June (a–i) and at the end of the experiment 6 weeks later on 21 July (j–r); sum of the relative biovolumes over seven nutrient enrichment levels (addition of 0, 1, 4, 9, 16, 25, and 36 μg P L^−1^) per browning level (a–c, j–l); generalized additive models for each browning level on logit‐transformed relative biovolumes of species within each of seven P enrichment levels (d–f, m–o); means and, in gray, 95% CIs (barely visible) of the first‐order derivatives across nutrient levels (g–i, p–r).

The frequency distribution of phytoplankton species covered a broad size range (Appendix S2: Figure S1), from picoplankton (e.g., *Cyanodictyon* spp., ~1 μm^3^) to the largest Dinophyceae (*Ceratium hirundinella*, ~50,000 μm^3^). In terms of biovolume, the distribution of species along the cell‐size gradient was less even (Appendix S2: Figure S2), as the total biovolume mostly comprised species of similar cell size, resulting in clustered distributions with biovolume peaks and troughs (Figure 2). Before the simulated storm, the size distribution was invariably unimodal (average of 7.7 log_2_ μm^3^ or 211 μm^3^) and dominated by small‐celled species (Figure 2a–i). Species in the 7 log_2_ μm^3^ size category (6–7 log_2_ μm^3^ equals 64–128 μm^3^) dominated the communities, contributing about 60% to the total phytoplankton biovolume in all enclosures (Figure 2d–f). Variance explained by the GAMs at the start of the experiment was 83%, 92%, and 95% in A, B, and C enclosures, respectively (Figure 2d–f, Appendix S2: Table S1). Cell size explained significant variation in species biovolumes in all three GAMs (all *p* < 0.001). No effect of nutrient enrichment was noted (all *p* > 0.10). The unimodal pattern was statistically supported by the first‐order derivatives of the GAMs, which were significantly positive (ascending portion of the peak) and negative (descending portion of the peak), as shown by the averages of the derivatives across nutrient levels (Figure 2g–i, Appendix S2: Table S1).

After the addition of cDOM and nutrients, the pattern shifted from unimodal to bimodal as the relative biovolume of larger species increased and that of smaller‐celled species decreased correspondingly (Figure 2j–r). Variance explained by the GAMs at the end of the experiment was 51%, 55%, and 72% in A, B, and C enclosures, respectively (Figure 2m–o, Appendix S2: Table S1). As observed at the start of the experiment, cell size explained significant variation in species biovolumes in all three GAMs (all *p* < 0.001), whereas an effect of nutrient enrichment was not apparent (all *p* > 0.40). At the end of the experiment, the size distribution was bimodal in virtually all enclosures (Figure 2m–r, Appendix S2: Figure S2), with the communities dominated by species in the 7 and 11 log_2_ μm^3^ size categories (64–128 μm^3^ and 1024–2048 μm^3^, respectively). However, the average (±SD) relative biovolume of species in the 11 log_2_ μm^3^ size category in the C (47 ± 20%) exceeded that in the B (28 ± 14%) and A enclosures (24 ± 18%). The bimodal pattern was statistically supported by the first‐order derivatives of the GAMs (Figure 2p–r), which were significantly positive (ascending portion of the peak) and negative (descending portion of the peak), respectively.

### Species richness

Phytoplankton species numbers declined in all enclosures during the course of the experiment (Appendix S3: Figure S1). Across all enclosures, an average of 39 ± 9% (±SD) of the species initially present was lost between the first and last day of the experiment, with losses in individual enclosures ranging between 18% and 54%. Species numbers significantly increased with increasing light availability (LME, *p* < 0.001; Appendix S3: Figure S2, Table S1). Mesozooplankton biomass was not significantly related to phytoplankton species numbers (LRT, χ^2^ = 0.96, *p* = 0.33). Likewise, there was no significant relationship between SRP and phytoplankton species numbers (LME, *p* = 0.79), nor was the interaction between PAR and SRP significantly related to the number of species (LME, *p* = 0.06; Appendix S3: Figure S2, Table S1). Including SRP concentration in addition to time in the model did not explain more of the temporal variation in species numbers in the clear A enclosures (LRT, χ^2^ < 0.1, *p* = 0.99; Appendix S3). Therefore and because of the negative correlation with PAR, SRP was not included in the PA model to minimize the number of variables. This allowed the model to converge.

**FIGURE 3:**
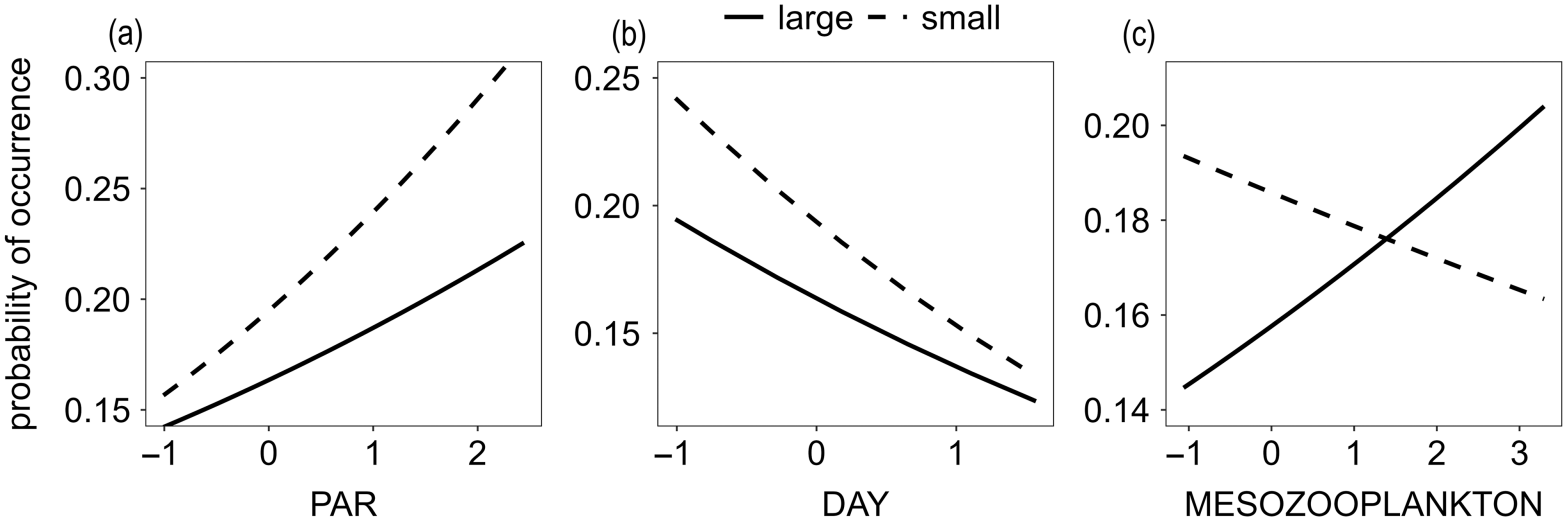
Estimated probability of species occurrences as a function of the interaction between cell‐size range (small‐celled vs. large‐celled species) and photosynthetically active radiation (PAR) (a), elapsed time (b), and zooplankton biomass (c) as standardized predictors.

At mean values of the standardized predictors PAR, elapsed time, and zooplankton biomass (the intercept, Table 1, Figure 3), the probability of species occurrence did not differ (*p* = 0.34) between the large (17% or −1.60 on the logit scale) and smaller species (20% or −1.36 on the logit scale). The PA model revealed a significant positive effect of PAR (*p* < 0.01, Figure 3, Table 1) on the probability of species occurrence of both size ranges, whereas elapsed time had a negative effect (*p* < 0.001). However, the size range appeared to have an effect on species occurrence along the gradient of mesozooplankton biomass (i.e., grazing pressure). The interaction between size range and zooplankton biomass was significant (*p* < 0.01), indicating that the occurrence of small species decreased relative to larger ones as zooplankton biomass increased. The occurrence of large phytoplankton species increased with increasing zooplankton biomass (*p* < 0.05).

**TABLE 1:** Statistics of the presence–absence generalized linear mixed model (logit scale; see Equation 1 in *Methods*), including SEs and bootstrapped 95% CIs (999 simulations) of the fixed factors.

Factor	Estimate	SE	95% CI	*p* value
β_0_	−1.60	0.19	−1.95; −1.24	<0.001
β_size_	0.24	0.25	−0.25; 0.66	0.34
β_PAR_	0.16	0.05	0.05; 0.28	<0.01
β_day_	−0.21	0.05	−0.32; −0.10	<0.001
β_zoo_	0.10	0.04	0.01; 0.18	<0.05
β_size×PAR_	0.10	0.07	−0.05; 0.25	0.15
β_size×day_	−0.07	0.06	−0.20; 0.06	0.25
β_size×zoo_	−0.14	0.05	−0.25; −0.03	<0.01

Abbreviation: PAR, photosynthetically active radiation.

### The biovolume models

**FIGURE 4:**
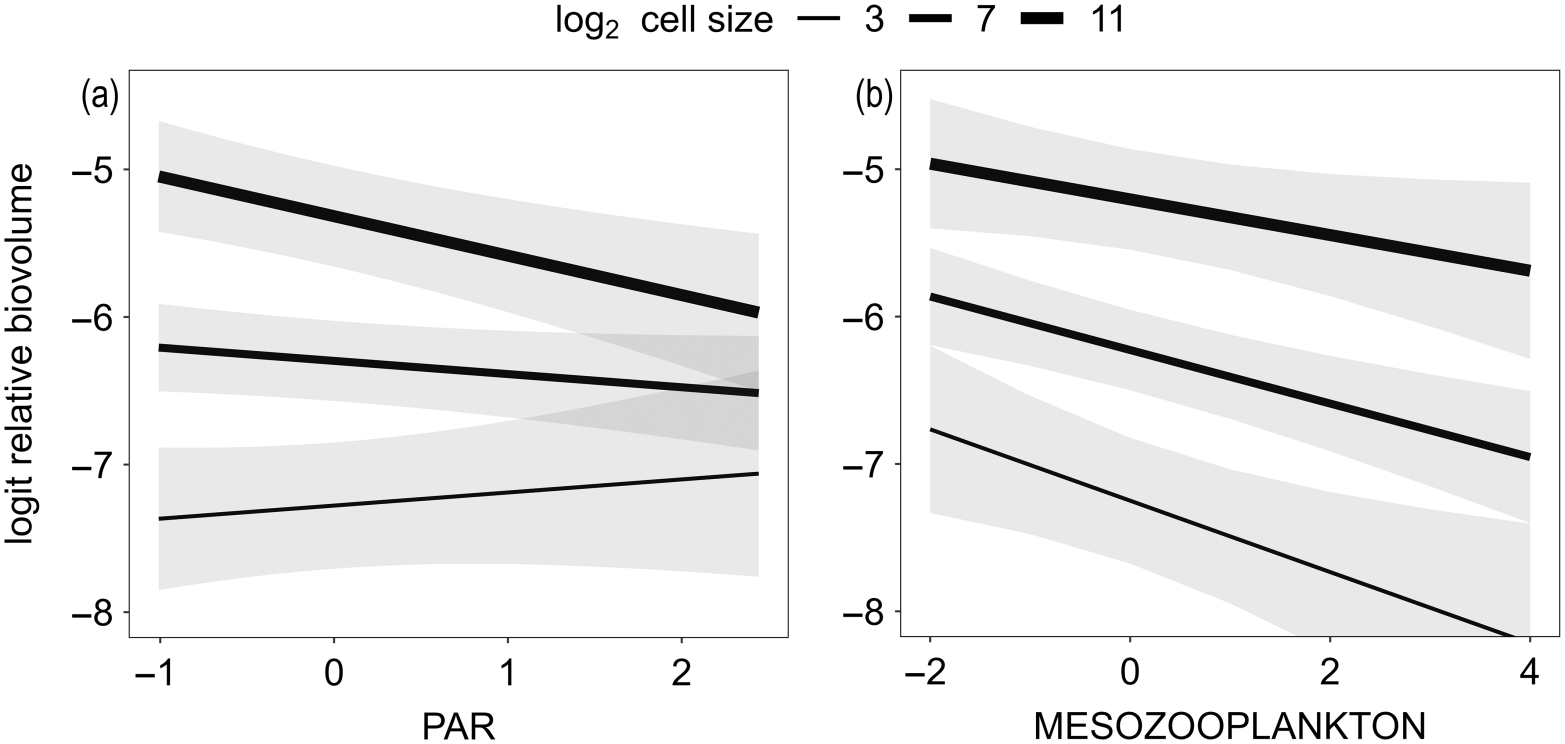
Predicted relationships between cell size and relative phytoplankton biovolume along gradients of standardized photosynthetically active radiation (PAR) (a) and zooplankton biomass (i.e., putative grazing pressure) (b). Bands represent 95% CIs. The three lines per plot indicate species with cell sizes of 3, 7, and 11 log_2_ μm^3^, corresponding to 8, 128, and 2048 μm^3^.

Phytoplankton responded to the PAR gradient in a species‐specific fashion, as shown by the comparison of the two biovolume models including random slopes for PAR or not (LRT, χ^2^ = 84.1, *p* < 0.001). Cell size had a significant effect on the relative biovolume of species along the PAR gradient (Figure 4a, β_size×PAR_ in Table 2), as shown by both LRT (χ^2^ = 4.3, *p* = 0.04) and parametric bootstrap (999 simulations, *p* = 0.04). The two biovolume peaks for the dominant species (cell size of 7 log_2_ μm^3^ or 128 μm^3^, and 11 log_2_ μm^3^ or 2048 μm^3^; Figure 2d–f,m–o) shown by the fit lines (Figure 4a) underline that the large‐celled species contributed more to the total community biovolume as PAR decreased by browning. In contrast, the total biovolume of the cluster comprising the smaller species (7 log_2_ μm^3^ or 128 μm^3^) showed little response to PAR. Species with 3 log_2_ μm^3^ cells (or 8 μm^3^) contributed significantly less to the biovolume than species with larger cells (*p* < 0.001) and tended to increase in biovolume as PAR increased (Figure 4a).

**TABLE 2:** Statistics of two linear mixed‐effects models (logit scale; see Equation 2 in *Methods*) describing the relationships between cell size and relative phytoplankton biovolume along gradients of standardized photosynthetically active radiation (PAR) and zooplankton biomass (i.e., putative grazing pressure).

Model	Factor	Estimate	SE	95% CI	*p* value
PAR	β_0_	−6.10	0.14	−6.34; −5.84	<0.001
	β_PAR_	−0.13	0.06	−0.24; −0.01	<0.05
	β_size_	0.64	0.09	0.46; 0.82	<0.001
	β_size×PAR_	−0.12	0.05	−0.22; −0.01	<0.05
Mesozooplankton biomass	β_0_	−6.02	0.14	−6.27; −5.75	<0.001
	β_zoo_	−0.17	0.04	−0.25; −0.08	<0.001
	β_size_	0.66	0.09	0.48; 0.84	<0.001
	β_size×zoo_	0.04	0.04	−0.04; 0.13	0.35

*Note*: 95% CI refers to the lower and upper 95% limits of the bootstrapped (999 simulations) CIs.

Phytoplankton also responded to the zooplankton biomass gradient in a species‐specific fashion (LRT, χ^2^ = 167.8, *p* < 0.001). Cell size had no significant effect on the relative biovolume of species along the gradient of zooplankton biomass (Figure 4b, nonsignificant mesozooplankton:cell‐size interaction, β_size×zoo_ in Table 2) as shown by both LRT (χ^2^ = 0.78, *p* = 0.38) and parametric bootstrap (999 simulations, *p* = 0.40). Nonetheless, phytoplankton biovolume significantly declined as zooplankton biomass increased (*p* < 0.001).

Cell size had no significant effect on the relative biovolume of species along the SRP gradient (Appendix S4: Figure S1a; LRT, χ^2^ = 3.68, *p* = 0.06; parametric bootstrap, 999 simulations, *p* = 0.07), nor over time (Appendix S4: Figure S1b; LRT, χ^2^ = 1.30, *p* = 0.25; parametric bootstrap, 999 simulations, *p* = 0.28).

## DISCUSSION

Our results indicating clear detrimental effects of lake browning on phytoplankton species richness and the probability of species occurrence support our first hypothesis, stating that phytoplankton species numbers will decrease with increased storm‐induced terrestrial runoff. This outcome is in line with results from previous small‐scale enclosure experiments (Rasconi et al., 2015; Urrutia‐Cordero et al., 2017) and appears to stem primarily from reduced primary production due to light limitation by cDOM (Cooney et al., 2018; Solomon et al., 2015). Apart from reduced irradiance levels, shifts in the underwater light spectrum may have played a role in causing these effects on species richness and occurrences because the preferential absorption of short‐wave PAR by cDOM could disrupt species coexistence due to niche partitioning along the PAR spectrum, which is enabled by distinct light‐harvesting complexes (Burson et al., 2019; Luimstra et al., 2020; Stomp et al., 2004). In support of this hypothesis, phytoplankton richness did indeed decline in a natural marine community exposed to red as opposed to white light (Hintz et al., 2021). Although not conclusive evidence that spectral partitioning enables species coexistence (Hintz et al., 2021), this mechanism, mediated by browning, likely had a bearing on species occurrence and richness in our experiment.

Browning can also indirectly affect phytoplankton communities, notably by altering nutrient availability, as indicated by elevated SRP and dissolved inorganic nitrogen (DIN) concentrations in our enclosures receiving cDOM. In particular, species characterized by high rates of nutrient uptake may outcompete others by capitalizing on nutrient pulses accompanying cDOM inputs (Sommer, 1984). Such nutrient effects are weakened when light limitation becomes overwhelming (Olson et al., 2020), as in our study. However, irrespective of the relative importance of direct versus indirect effects, our results indicate that altered light conditions due to browning lead to notable shifts in phytoplankton communities if precipitation and runoff intensify in the future, as predicted (IPCC, 2023).

The most striking outcome of our analysis of phytoplankton communities under varying environmental conditions is the clustering of species in two distinct size classes along the cell‐size gradient. Such a bimodal (or even multimodal) distribution has been observed in communities ranging from beetles (Scheffer & Van Nes, 2006) to birds (Thibault et al., 2011) and mammals (Holling, 1992), and from trees (D'Andrea et al., 2020) to phytoplankton (Downing et al., 2014; Segura et al., 2011, 2013; Vergnon et al., 2009, 2012). This begs the question whether or not the pattern is driven by general ecological processes. Possible drivers are stabilizing niche differences (i.e., niche partitioning) and equalizing mechanisms reducing fitness differences among competing species (i.e., neutrality; Adler et al., 2007; Chesson, 2000). Both could also act in tandem (Downing et al., 2014; Segura et al., 2011, 2013; Vergnon et al., 2009, 2012), as articulated in a concept known as emergent neutrality or self‐organized similarity (Holt, 2006; Scheffer et al., 2018; Scheffer & Van Nes, 2006). Accordingly, species can coexist if differences between or among clusters are sufficiently large to limit competition, and at the same time sufficiently similar within clusters to prevent competitive exclusion. Both forces could have contributed to the shift from the unimodal to the bimodal cell‐size distribution emerging during our experiment, supporting the idea of emergent neutrality, whereas Hutchinson's paradox arose, in part, because of his unique focus on niche differences. This conclusion also matches results from a laboratory experiment assessing competitive outcomes in pairs of resident versus invading cyanobacteria species differing in cell or colony size, showing that although size differences were insufficient to predict species coexistence, both niche and fitness differences increased with size differences (Gallego et al., 2019).

Despite emerging as a general pattern in our experiment, the clustering we observed played out differently across experimental treatments. Specifically, the cluster of smaller‐sized cells was dominant in clear water, whereas the cluster comprising larger cells assumed greater importance at elevated levels of browning. This difference was counter to our expectations according to Hypothesis 2, which stated that the size distribution of species under conditions of low light and high nutrient availability would shift toward small cells because light‐use traits scaling with cell size favor small‐celled species under dim light (Edwards et al., 2015; Schwaderer et al., 2011). Several processes could have led to the unexpected outcome.

Firstly, since the maximum nutrient quota (*Q*_max_) increases more strongly with cell size than the minimum quota (*Q*_min_), large species have a greater storage capacity (*Q*_max_/*Q*_min_) (Hillebrand et al., 2022; Litchman et al., 2009), facilitating luxury P uptake (Solovchenko et al., 2019). This confers them a competitive advantage under fluctuating nutrient conditions because they can benefit from cellular nutrient pools built up earlier to sustain growth during exposure to cDOM when SRP and DIN uptake appear to be impaired at the low light levels in our experiment.

Secondly, cell size regulates resource acquisition, shifting the trophic function from small autotrophic species to intermediate mixotrophic species to large heterotrophic species (Ward & Follows, 2016). Although the size distribution was bimodal in virtually all our enclosures, it is likely that browning by cDOM exacerbated the development of large mixotrophic species, as reported in other browning enclosure studies (e.g., Urrutia‐Cordero et al., 2017).

Thirdly, despite the importance of cell size as a master trait (Finkel et al., 2010; Irwin et al., 2006; Key et al., 2010), other species traits such as motility, buoyancy, or colony formation (Clegg et al., 2003; Litchman & Klausmeier, 2008) may also influence resource acquisition and hence the cell‐size distribution of phytoplankton. For example, colonies of cyanobacteria avoid increased self‐shading with increasing size by decreasing cellular pigment concentrations or the cell density in colonies (Agustí & Phlips, 1992). This would be particularly important when increasing colony size strengthens predator avoidance, as seen by the 10‐fold increase in mean colony diameter by *Microcystis aeruginosa* exposed to grazing pressure by the flagellate *Ochromonas* sp. (Yang & Kong, 2012).

Lastly, the plasticity of species traits relating to resource use may play a role, particularly the ability of species to acclimate to changing light conditions (Dubinsky & Stambler, 2009; Guislain et al., 2019). Here, spectral composition may be important as well, in addition to fluctuating irradiance levels (Hintz et al., 2022), with chromatic acclimation predicted to be beneficial in environments where red and green light of the PAR spectrum predominate (Sanfilippo et al., 2019). Green light typically prevails in lakes such as Lake Stechlin, while strong light absorption by cDOM, especially in the short‐wave range, moves the spectrum toward longer wavelengths (red light). Although chromatic acclimation mostly involves the adjustment of chromophores (Kehoe & Gutu, 2006; Richardson, 2022; Sanfilippo et al., 2019), cell morphology can also change, as described for a filamentous cyanobacterium, *Fremyella diplosiphon*, where cylindrical and smaller cells grown under green light become larger and rounder when exposed to red light. The underlying reason is unknown (Gutu & Kehoe, 2012), but the phenomenon implies that some larger cells may have a competitive advantage over others under red‐shifted light resulting from lake browning.

Our results also provide insights into the importance of cell size on the dominance structure (evenness) of communities and highlight the need to determine trait distributions within communities to predict community response to environmental change (Hillebrand et al., 2008; Norberg, 2004; Soininen et al., 2012). Indeed, the size‐dependent effect of PAR that we found in our biovolume analysis, but not on species occurrence, contrasts with the apparent size‐dependent influence of mesozooplankton grazing on the probability of species occurrence, but not on biovolumes. In discordance with our Hypothesis 3 (stating that large cells will increase in abundance at elevated light and nutrient levels because of sufficient access to both resources and a lower grazing mortality), these differences are likely due to the tendency of rare species with a low biovolume to go undetected at low light levels. Additionally, a role may be played by a greater susceptibility of small species to grazing without notably altering the cell‐size distribution of the relative biovolumes of species in the communities.

Overall, our findings offer mixed support for our initial hypotheses, revealing complex size‐related dynamics in phytoplankton communities influenced by lake browning. Hypothesis 1, which predicts a decline in species richness with increased terrestrial runoff, was supported because the probability of species occurrence decreased under browning. In contrast, Hypothesis 2 was not supported. Instead of favoring small‐celled species under low light and high nutrient availability, as we had hypothesized, browning led to a stronger dominance of larger cells. This suggests that traits other than light‐use efficiency were important in ensuring the success of larger species in these conditions. Traits such as nutrient storage or mixotrophy could be instrumental in this respect. Based on Hypothesis 3, we anticipated an increase in the abundance of large cells under high light and nutrient levels due to lower grazing mortality. However, our results indicate that, although the occurrence of smaller species was indeed more susceptible to grazing, the effect was not strong enough to notably alter the cell‐size distribution of species in the communities.

In conclusion, the results of our experiment conducted in large enclosures designed to mimic realistic environmental conditions point to a distinct competitive advantage of larger over smaller phytoplankton species under reduced light availability caused by lake browning. This underlines that cell size is a critically important species trait influencing the structure and dynamics of phytoplankton communities after storm events carrying cDOM to clearwater lakes. What is more, the observed clustering of species in distinct size classes along the cell‐size gradient implies that size differences among species may matter as much as the size of species per se to understand phytoplankton dynamics. Given that shifts in cell‐size distribution have implications for food‐web structure and biogeochemical cycles, our results thus highlight the importance of determining phytoplankton size structure as an essential element to forecast lake community and ecosystem dynamics in response to environmental change.

## AUTHOR CONTRIBUTIONS

Alexis L. N. Guislain conceived the study and analyzed the data. Mark O. Gessner, Anne Lyche Solheim, Stella A. Berger, Jens C. Nejstgaard, Ute Mischke, and Hans‐Peter Grossart designed the experiment, which was coordinated by Stella A. Berger and Jens C. Nejstgaard. Jens C. Nejstgaard and Stella A. Berger designed, constructed, and tested the technical setup enabling the large‐scale filling and mixing of the experiment. All authors collected data. Ute Mischke and Birger Skjelbred identified and counted phytoplankton and determined their biomass. Erik Sperfeld identified and counted zooplankton and determined its biomass. Alexis L. N. Guislain wrote the manuscript with major contributions from Mark O. Gessner, Jens C. Nejstgaard, Jan Köhler, Erik Sperfeld, Hans‐Peter Grossart, Anne Lyche Solheim, and Stella A. Berger. The manuscript was approved by all authors.

## CONFLICT OF INTEREST STATEMENT

The authors declare no conflicts of interest.

## Supporting information


Appendix S1.

Appendix S2.

Appendix S3.

Appendix S4.

## Data Availability

Data are available in the Freshwater Research and Environmental Database (FRED) at https://doi.org/10.18728/igb-fred-951.0 (Berger et al., 2025) maintained by the Leibniz Institute of Freshwater Ecology and Inland Fisheries (IGB).

## References

[ecy70043-bib-0001] Adler, P. B. , J. HilleRisLambers , and J. M. Levine . 2007. “A Niche for Neutrality.” Ecology Letters 10: 95–104. 17257097 10.1111/j.1461-0248.2006.00996.x

[ecy70043-bib-0002] Agustí, S. , and E. J. Phlips . 1992. “Light Absorption by Cyanobacteria: Implications of the Colonial Growth Form.” Limnology and Oceanography 37: 434–441.

[ecy70043-bib-0003] Bates, D. , M. Mächler , B. Bolker , and S. Walker . 2015. “Fitting Linear Mixed‐Effects Models Using {lme4}.” Journal of Statistical Software 67: 1–48.

[ecy70043-bib-0004] Berger, S. A. , S. Diehl , H. Stibor , P. Sebastian , and A. Scherz . 2014. “Separating Effects of Climatic Drivers and Biotic Feedbacks on Seasonal Plankton Dynamics: no Sign of Trophic Mismatch.” Freshwater Biology 59: 2204–2220.

[ecy70043-bib-0005] Berger, S. A. , A. Lyche Solheim , J. C. Nejstgaard , U. Beyer , M. Degebrodt , E. Huth , M. Lentz , et al. 2025. “Large Enclosure Experiment (LakeLab in Lake Stechlin) to Study Effects of Browning and Nutrient Loading on Lake Plankton: Light, Nutrients, Phytoplankton Cell Size, and Community Structure.” IGB Leibniz‐Institute of Freshwater Ecology and Inland Fisheries. 10.18728/igb-fred-951.0.

[ecy70043-bib-0006] Bottrell, H. H. , A. Duncan , Z. M. Gliwicz , E. Grygierek , A. Herzig , A. Hillbricht‐Ilkowska , H. Kurasawa , P. Larsson , and T. Weglenska . 1976. “A Review of some Problems in Zooplankton Production Studies.” Norwegian Journal of Zoology 24: 419–456.

[ecy70043-bib-0007] Brauer, V. S. , M. Stomp , and J. Huisman . 2012. “The Nutrient‐Load Hypothesis: Patterns of Resource Limitation and Community Structure Driven by Competition for Nutrients and Light.” American Naturalist 179: 721–740. 10.1086/665650 22617261

[ecy70043-bib-0008] Burson, A. , M. Stomp , L. Mekkes , and J. Huisman . 2019. “Stable Coexistence of Equivalent Nutrient Competitors through Niche Differentiation in the Light Spectrum.” Ecology 100: 1–12. 10.1002/ecy.2873 PMC6916172 31463935

[ecy70043-bib-0009] CEN . 2015. EN 16695: Water Quality – Guidance on the Estimation of Phytoplankton Biovolume. Brussels: European Committee for Standardization.

[ecy70043-bib-0010] Chang, F. H. , E. C. Marquis , C. W. Chang , G. C. Gong , and C. H. Hsieh . 2013. “Scaling of Growth Rate and Mortality with Size and its Consequence on Size Spectra of Natural Microphytoplankton Assemblages in the East China Sea.” Biogeosciences 10: 5267–5280.

[ecy70043-bib-0011] Chesson, P. 2000. “Mechanisms of Maintenance of Species Diversity.” Annual Review of Ecology and Systematics 31: 343–366.

[ecy70043-bib-0012] Clegg, M. R. , S. C. Maberly , and R. I. Jones . 2003. “The Effect of Photon Irradiance on the Behavioral Ecology and Potential Niche Separation of Freshwater Phytoplanktonic Flagellates.” Journal of Phycology 39: 650–662.

[ecy70043-bib-0013] Cooney, E. M. , P. McKinney , R. Sterner , G. E. Small , and E. C. Minor . 2018. “Tale of Two Storms: Impact of Extreme Rain Events on the Biogeochemistry of Lake Superior.” Journal of Geophysical Research – Biogeosciences 123: 1719–1731. 30800557 10.1029/2017JG004216 PMC6381994

[ecy70043-bib-0014] Cunningham, R. B. , and D. B. Lindenmayer . 2005. “Modeling Count Data of Rare Species: Some Statistical Issues.” Ecology 86: 1135–1142.

[ecy70043-bib-0015] D'Andrea, R. , J. Guittar , J. P. O'Dwyer , H. Figueroa , S. J. Wright , R. Condit , and A. Ostling . 2020. “Counting Niches: Abundance‐by‐Trait Patterns Reveal Niche Partitioning in a Neotropical Forest.” Ecology 101: 1–9. 10.1002/ecy.3019 32078155

[ecy70043-bib-0016] Downing, A. S. , S. Hajdu , O. Hjerne , S. A. Otto , T. Blenckner , U. Larsson , and M. Winder . 2014. “Zooming in on Size Distribution Patterns Underlying Species Coexistence in Baltic Sea Phytoplankton.” Ecology Letters 17: 1219–1227. 25040569 10.1111/ele.12327

[ecy70043-bib-0017] Drakare, S. , P. Blomqvist , A. K. Bergström , and M. Jansson . 2002. “Primary Production and Phytoplankton Composition in Relation to DOC Input and Bacterioplankton Production in Humic Lake Örträsket.” Freshwater Biology 47: 41–52.

[ecy70043-bib-0018] Dubinsky, Z. , and N. Stambler . 2009. “Photoacclimation Processes in Phytoplankton: Mechanisms, Consequences, and Applications.” Aquatic Microbial Ecology 56: 163–176.

[ecy70043-bib-0019] Edwards, K. F. , E. Litchman , and C. A. Klausmeier . 2013a. “Functional Traits Explain Phytoplankton Community Structure and Seasonal Dynamics in a Marine Ecosystem.” Ecology Letters 16: 56–63. 23033839 10.1111/ele.12012

[ecy70043-bib-0020] Edwards, K. F. , E. Litchman , and C. A. Klausmeier . 2013b. “Functional Traits Explain Phytoplankton Responses to Environmental Gradients across Lakes of the United States.” Ecology 94: 1626–1635. 23951722 10.1890/12-1459.1

[ecy70043-bib-0021] Edwards, K. F. , M. K. Thomas , C. A. Klausmeier , and E. Litchman . 2012. “Allometric Scaling and Taxonomic Variation in Nutrient Utilization Traits and Maximum Growth Rate of Phytoplankton.” Limnology and Oceanography 57: 554–566.

[ecy70043-bib-0022] Edwards, K. F. , M. K. Thomas , C. A. Klausmeier , and E. Litchman . 2015. “Light and Growth in Marine Phytoplankton: Allometric, Taxonomic, and Environmental Variation.” Limnology and Oceanography 60: 540–552.

[ecy70043-bib-0023] Finkel, Z. V. , J. Beardall , K. J. Flynn , A. Quigg , T. A. V. Rees , and J. A. Raven . 2010. “Phytoplankton in a Changing World: Cell Size and Elemental Stoichiometry.” Journal of Plankton Research 32: 119–137.

[ecy70043-bib-0024] Fonvielle, J. A. , D. P. Giling , T. Dittmar , S. A. Berger , J. C. Nejstgaard , A. Lyche Solheim , M. O. Gessner , H.‐P. Grossart , and G. Singer . 2021. “Exploring the Suitability of Ecosystem Metabolomes to Assess Imprints of Brownification and Nutrient Enrichment on Lakes.” Journal of Geophysical Research – Biogeosciences 126: e2020JG005903.

[ecy70043-bib-0025] Gallego, I. , P. Venail , and B. W. Ibelings . 2019. “Size Differences Predict Niche and Relative Fitness Differences between Phytoplankton Species but Not their Coexistence.” ISME Journal 13: 1133–1143. 30607028 10.1038/s41396-018-0330-7 PMC6474302

[ecy70043-bib-0026] Gause, G. F. 1934. The Struggle for Existence. New York: Hafner Publishing. 10.1126/science.79.2036.16-a 17821472

[ecy70043-bib-0027] Giling, D. P. , J. C. Nejstgaard , S. A. Berger , H.‐P. Grossart , G. Kirillin , A. Penske , M. Lentz , P. Casper , J. Sareyka , and M. O. Gessner . 2017. “Thermocline Deepening Boosts Ecosystem Metabolism: Evidence from a Large‐Scale Lake Enclosure Experiment Simulating a Summer Storm.” Global Change Biology 23: 1448–1462. 27664076 10.1111/gcb.13512

[ecy70043-bib-0028] Guislain, A. , B. E. Beisner , and J. Köhler . 2019. “Variation in Species Light Acquisition Traits under Fluctuating Light Regimes: Implications for Non‐equilibrium Coexistence.” Oikos 128: 716–728.

[ecy70043-bib-0029] Gutu, A. , and D. M. Kehoe . 2012. “Emerging Perspectives on the Mechanisms, Regulation, and Distribution of Light Color Acclimation in Cyanobacteria.” Molecular Plant 5: 1–13. 21772031 10.1093/mp/ssr054

[ecy70043-bib-0030] Hautier, Y. , P. A. Niklaus , and A. Hector . 2009. “Competition for Light Causes Plant Biodiversity Loss after Eutrophication.” Science 324: 636–638. 19407202 10.1126/science.1169640

[ecy70043-bib-0031] Hillebrand, H. , E. Acevedo‐Trejos , S. D. Moorthi , A. Ryabov , M. Striebel , P. K. Thomas , and M. L. Schneider . 2022. “Cell Size as Driver and Sentinel of Phytoplankton Community Structure and Functioning.” Functional Ecology 36: 276–293.

[ecy70043-bib-0032] Hillebrand, H. , D. M. Bennett , and M. W. Cadotte . 2008. “Consequences of Dominance: A Review of Evenness Effects on Local and Regional Ecosystem Processes.” Ecology 89: 1510–1520. 18589516 10.1890/07-1053.1

[ecy70043-bib-0033] Hintz, N. H. , B. Schulze , A. Wacker , and M. Striebel . 2022. “Ecological Impacts of Photosynthetic Light Harvesting in Changing Aquatic Environments: A Systematic Literature Map.” Ecology and Evolution 12: 1–16. 10.1002/ece3.8753 PMC8939368 35356568

[ecy70043-bib-0034] Hintz, N. H. , M. Zeising , and M. Striebel . 2021. “Changes in Spectral Quality of Underwater Light Alter Phytoplankton Community Composition.” Limnology and Oceanography 66: 3327–3337.

[ecy70043-bib-0035] Holling, C. S. 1992. “Cross‐Scale Morphology, Geometry, and Dynamics of Ecosystems.” Ecological Monographs 62: 447–502.

[ecy70043-bib-0036] Holt, R. D. 2006. “Emergent neutrality.” Trends in Ecology & Evolution 21: 531–533. 16901580 10.1016/j.tree.2006.08.003

[ecy70043-bib-0037] Hubbell, S. P. 2001. The Unified Neutral Theory of Biodiversity and Biogeography. Princeton, NJ: Princeton University Press.

[ecy70043-bib-0038] Hutchinson, G. E. 1961. “The Paradox of the Plankton.” The American Naturalist 95: 137–145.

[ecy70043-bib-0039] Intergovernmental Panel on Climate Change (IPCC) . 2023. “Technical Summary.” In Climate Change 2021: The Physical Science Basis. Working Group I Contribution to the Sixth Assessment Report of the Intergovernmental Panel on Climate Change, 35–144. Cambridge and New York: Cambridge University Press.

[ecy70043-bib-0040] Irwin, A. J. , Z. V. Finkel , O. M. E. Schofield , and P. G. Falkowski . 2006. “Scaling‐up from Nutrient Physiology to the Size‐Structure of Phytoplankton Communities.” Journal of Plankton Research 28: 459–471.

[ecy70043-bib-0041] Jennings, E. , S. Jones , L. Arvola , P. A. Staehr , E. Gaiser , I. D. Jones , K. C. Weathers , G. A. Weyhenmeyer , C. Y. Chiu , and E. De Eyto . 2012. “Effects of Weather‐Related Episodic Events in Lakes: An Analysis Based on High‐Frequency Data.” Freshwater Biology 57: 589–601.

[ecy70043-bib-0042] Kasprzak, P. 1983. “Struktur, Populationsdynamik Und Assimilationsleistung Des Crustaceenplanktons Im Oligotrophen Stechlinsee.” PhD diss., Universität Dresden.

[ecy70043-bib-0043] Kasprzak, P. , T. Shatwell , M. O. Gessner , T. Gonsiorczyk , G. Kirillin , G. Selmeczy , J. Padisák , and C. Engelhardt . 2017. “Extreme Weather Event Triggers Cascade towards Extreme Turbidity in a Clear‐Water Lake.” Ecosystems 20: 1407–1420.

[ecy70043-bib-0044] Kehoe, D. M. , and A. Gutu . 2006. “Responding to Color: The Regulation of Complementary Chromatic Adaptation.” Annual Review of Plant Biology 57: 127–150. 10.1146/annurev.arplant.57.032905.105215 16669758

[ecy70043-bib-0045] Kelly, P. T. , C. T. Solomon , B. C. Weidel , and S. E. Jones . 2014. “Terrestrial Carbon Is a Resource, but Not a Subsidy, for Lake Zooplankton.” Ecology 95: 1236–1242. 25000755 10.1890/13-1586.1

[ecy70043-bib-0046] Key, T. , A. McCarthy , D. A. Campbell , C. Six , S. Roy , and Z. V. Finkel . 2010. “Cell Size Trade‐Offs Govern Light Exploitation Strategies in Marine Phytoplankton.” Environmental Microbiology 12: 95–104. 19735282 10.1111/j.1462-2920.2009.02046.x

[ecy70043-bib-0047] Kirk, J. T. 1994. Light and Photosynthesis in Aquatic Ecosystems. Cambridge: Cambridge University Press.

[ecy70043-bib-0048] Kritzberg, E. S. , W. Granéli , J. Björk , C. Brönmark , P. Hallgren , A. Nicolle , A. Persson , and L. A. Hansson . 2014. “Warming and Browning of Lakes: Consequences for Pelagic Carbon Metabolism and Sediment Delivery.” Freshwater Biology 59: 325–336.

[ecy70043-bib-0049] Litchman, E. 2022. “Understanding and Predicting Harmful Algal Blooms in a Changing Climate: A Trait‐Based Framework.” Limnology and Oceanography Letters 8: 229–246.

[ecy70043-bib-0050] Litchman, E. , and C. A. Klausmeier . 2008. “Trait‐Based Community Ecology of Phytoplankton.” Annual Review of Ecology, Evolution, and Systematics 39: 615–639.

[ecy70043-bib-0051] Litchman, E. , C. A. Klausmeier , and K. Yoshiyama . 2009. “Contrasting Size Evolution in Marine and Freshwater Diatoms.” Proceedings of the National Academy of Sciences of the United States of America 106: 2665–2670. 19202058 10.1073/pnas.0810891106 PMC2650323

[ecy70043-bib-0052] Lüdecke, D. , M. S. Ben‐Shachar , I. Patil , P. Waggoner , and D. Makowski . 2021. “{performance}: An {R} Package for Assessment, Comparison and Testing of Statistical Models.” Journal of Open Source Software 6: 3139.

[ecy70043-bib-0053] Luimstra, V. M. , J. M. H. Verspagen , T. Xu , J. M. Schuurmans , and J. Huisman . 2020. “Changes in Water Color Shift Competition between Phytoplankton Species with Contrasting Light‐Harvesting Strategies.” Ecology 101: 1–16. 10.1002/ecy.2951 PMC7079016 31840230

[ecy70043-bib-0054] Lyche Solheim, A. , H. Gundersen , U. Mischke , B. Skjelbred , J. C. Nejstgaard , A. L. N. Guislain , E. Sperfeld , et al. 2024. “Lake Browning Counteracts Cyanobacteria Responses to Nutrients: Evidence from Phytoplankton Dynamics in Large Enclosure Experiments and Comprehensive Observational Data.” Global Change Biology 30: 1–23. 10.1111/gcb.17013 37994377

[ecy70043-bib-0055] MacArthur, R. , and R. Levins . 1967. “The Limiting Similarity, Convergence, and Divergence of Coexisting Species.” The American Naturalist 101: 377–385.

[ecy70043-bib-0056] Marañón, E. 2015. “Cell Size as a Key Determinant of Phytoplankton Metabolism and Community Structure.” Annual Review of Marine Science 7: 241–264. 10.1146/annurev-marine-010814-015955 25062405

[ecy70043-bib-0057] Marañón, E. , P. Cermeño , D. C. López‐Sandoval , T. Rodríguez‐Ramos , C. Sobrino , M. Huete‐Ortega , J. M. Blanco , and J. Rodríguez . 2013. “Unimodal Size Scaling of Phytoplankton Growth and the Size Dependence of Nutrient Uptake and Use.” Ecology Letters 16: 371–379. 23279624 10.1111/ele.12052

[ecy70043-bib-0058] May, R. M. 1988. “How Many Species Are There on Earth?” Science 241: 1441–1449. 17790039 10.1126/science.241.4872.1441

[ecy70043-bib-0059] McGill, B. J. , B. J. Enquist , E. Weiher , and M. Westoby . 2006. “Rebuilding Community Ecology from Functional Traits.” Trends in Ecology & Evolution 21: 178–185. 16701083 10.1016/j.tree.2006.02.002

[ecy70043-bib-0060] Minguez, L. , E. Sperfeld , S. A. Berger , J. C. Nejstgaard , and M. O. Gessner . 2020. “Changes in Food Characteristics Reveal Indirect Effects of Lake Browning on Zooplankton Performance.” Limnology and Oceanography 65: 1028–1040.

[ecy70043-bib-0061] Mora, C. , D. P. Tittensor , S. Adl , A. G. B. Simpson , and B. Worm . 2011. “How Many Species Are There on Earth and in the Ocean?” PLoS Biology 9: 1–8. 10.1371/journal.pbio.1001127 PMC3160336 21886479

[ecy70043-bib-0062] Mutshinda, C. M. , A. Mishra , Z. V. Finkel , C. E. Widdicombe , and A. J. Irwin . 2022. “Bayesian Two‐Part Modeling of Phytoplankton Biomass and Occurrence.” Hydrobiologia 849: 1287–1300.

[ecy70043-bib-0063] Norberg, J. 2004. “Biodiversity and Ecosystem Functioning: A Complex Adaptive Systems Approach.” Limnology and Oceanography 49: 1269–1277.

[ecy70043-bib-0064] Olson, C. R. , C. T. Solomon , and S. E. Jones . 2020. “Shifting Limitation of Primary Production: Experimental Support for a New Model in Lake Ecosystems.” Ecology Letters 23: 1800–1808. 32945617 10.1111/ele.13606 PMC7756323

[ecy70043-bib-0065] Pančić, M. , and T. Kiørboe . 2018. “Phytoplankton Defence Mechanisms: Traits and Trade‐Offs.” Biological Reviews 93: 1269–1303. 29356270 10.1111/brv.12395

[ecy70043-bib-0066] Ptacnik, R. , S. D. Moorthi , and H. Hillebrand . 2010. “Hutchinson Reversed, or Why There Need to Be So Many Species.” Advances in Ecological Research 43: 1–43.

[ecy70043-bib-0067] R Core Team . 2022. R: A Language and Environment for Statistical Computing. Vienna: R Foundation for Statistical Computing.

[ecy70043-bib-0068] Rasconi, S. , A. Gall , K. Winter , and M. J. Kainz . 2015. “Increasing Water Temperature Triggers Dominance of Small Freshwater Plankton.” PLoS One 10: 1–17. 10.1371/journal.pone.0140449 PMC4603799 26461029

[ecy70043-bib-0069] Richardson, T. L. 2022. “The Colorful World of Cryptophyte Phycobiliproteins.” Journal of Plankton Research 44: 814–826.

[ecy70043-bib-0070] Sanfilippo, J. E. , L. Garczarek , F. Partensky , and D. M. Kehoe . 2019. “Chromatic Acclimation in Cyanobacteria: A Diverse and Widespread Process for Optimizing Photosynthesis.” Annual Review of Microbiology 73: 407–433. 10.1146/annurev-micro-020518-115738 31500538

[ecy70043-bib-0071] Scharnweber, K. , S. Peura , K. Attermeyer , S. Bertilsson , L. Bolender , M. Buck , K. Einarsdóttir , et al. 2021. “Comprehensive Analysis of Chemical and Biological Problems Associated with Browning Agents Used in Aquatic Studies.” Limnology and Oceanography: Methods 19: 818–835.

[ecy70043-bib-0072] Scheffer, M. , and E. H. Van Nes . 2006. “Self‐Organized Similarity, the Evolutionary Emergence of Groups of Similar Species.” Proceedings of the National Academy of Sciences of the United States of America 103: 6230–6235. 16585519 10.1073/pnas.0508024103 PMC1458860

[ecy70043-bib-0073] Scheffer, M. , E. H. Van Nes , and R. Vergnon . 2018. “Toward a Unifying Theory of Biodiversity.” Proceedings of the National Academy of Sciences of the United States of America 115: 639–641. 29326234 10.1073/pnas.1721114115 PMC5789964

[ecy70043-bib-0074] Schwaderer, A. S. , K. Yoshiyama , P. De Tezanos Pinto , N. G. Swenson , C. A. Klausmeier , and E. Litchman . 2011. “Eco‐Evolutionary Differences in Light Utilization Traits and Distributions of Freshwater Phytoplankton.” Limnology and Oceanography 56: 589–598.

[ecy70043-bib-0075] Segura, A. M. , D. Calliari , C. Kruk , D. Conde , S. Bonilla , and H. Fort . 2011. “Emergent Neutrality Drives Phytoplankton Species Coexistence.” Proceedings of the Royal Society B: Biological Sciences 278: 2355–2361. 10.1098/rspb.2010.2464 PMC3119015 21177680

[ecy70043-bib-0076] Segura, A. M. , C. Kruk , D. Calliari , F. García‐Rodriguez , D. Conde , C. E. Widdicombe , and H. Fort . 2013. “Competition Drives Clumpy Species Coexistence in Estuarine Phytoplankton.” Scientific Reports 3: 1–6. 10.1038/srep01037 PMC3539148 23301158

[ecy70043-bib-0077] Simpson, G. L. 2023. “gratia: Graceful ggplot‐Based Graphics and Other Functions for GAMs Fitted using mgcv.” https://gavinsimpson.github.io/gratia/.

[ecy70043-bib-0078] Soininen, J. , S. Passy , and H. Hillebrand . 2012. “The Relationship between Species Richness and Evenness: A Meta‐Analysis of Studies across Aquatic Ecosystems.” Oecologia 169: 803–809. 22210185 10.1007/s00442-011-2236-1

[ecy70043-bib-0079] Solomon, C. T. , S. E. Jones , B. C. Weidel , I. Buffam , M. L. Fork , J. Karlsson , S. Larsen , et al. 2015. “Ecosystem Consequences of Changing Inputs of Terrestrial Dissolved Organic Matter to Lakes: Current Knowledge and Future Challenges.” Ecosystems 18: 376–389.

[ecy70043-bib-0080] Solovchenko, A. E. , T. T. Ismagulova , A. A. Lukyanov , S. G. Vasilieva , I. V. Konyukhov , S. I. Pogosyan , E. S. Lobakova , and O. A. Gorelova . 2019. “Luxury Phosphorus Uptake in Microalgae.” Journal of Applied Phycology 3: 2755–2770.

[ecy70043-bib-0081] Sommer, U. 1984. “The Paradox of the Plankton: Fluctuations of Phosphorus Availability Maintain Diversity of Phytoplankton in Flow‐Through Cultures.” Limnology and Oceanography 29: 633–636.

[ecy70043-bib-0082] Sterner, R. W. , J. J. Elser , E. J. Fee , S. J. Guildford , and T. H. Chrzanowski . 1997. “The Light:Nutrient Ratio in Lakes: The Balance of Energy and Materials Affects Ecosystem Structure and Process.” American Naturalist 150: 663–684. 10.1086/286088 18811330

[ecy70043-bib-0083] Stockwell, J. D. , J. P. Doubek , R. Adrian , O. Anneville , C. C. Carey , L. Carvalho , L. N. De Senerpont Domis , et al. 2020. “Storm Impacts on Phytoplankton Community Dynamics in Lakes.” Global Change Biology 26: 2756–2784. 32133744 10.1111/gcb.15033 PMC7216882

[ecy70043-bib-0084] Stomp, M. , J. Huisman , F. De Jongh , A. J. Veraart , D. Gerla , M. Rijkeboer , B. W. Ibelings , U. I. A. Wollenzien , and L. J. Stal . 2004. “Adaptive Divergence in Pigment Composition Promotes Phytoplankton Biodiversity.” Nature 432: 104–107. 15475947 10.1038/nature03044

[ecy70043-bib-0085] Thibault, K. M. , E. P. White , A. H. Hurlbert , and S. K. M. Ernest . 2011. “Multimodality in the Individual Size Distributions of Bird Communities.” Global Ecology and Biogeography 20: 145–153.

[ecy70043-bib-0086] Thrane, J. E. , D. O. Hessen , and T. Andersen . 2014. “The Absorption of Light in Lakes: Negative Impact of Dissolved Organic Carbon on Primary Productivity.” Ecosystems 17: 1040–1052.

[ecy70043-bib-0087] Urrutia‐Cordero, P. , M. K. Ekvall , J. Ratcovich , M. Soares , S. Wilken , H. Zhang , and L. A. Hansson . 2017. “Phytoplankton Diversity Loss along a Gradient of Future Warming and Brownification in Freshwater Mesocosms.” Freshwater Biology 62: 1869–1878.

[ecy70043-bib-0088] Vergnon, R. , N. K. Dulvy , and R. P. Freckleton . 2009. “Niches Versus Neutrality: Uncovering the Drivers of Diversity in a Species‐Rich Community.” Ecology Letters 12: 1079–1090. 19747181 10.1111/j.1461-0248.2009.01364.x

[ecy70043-bib-0089] Vergnon, R. , E. H. Van Nes , and M. Scheffer . 2012. “Emergent Neutrality Leads to Multimodal Species Abundance Distributions.” Nature Communications 3: 1–6. 10.1038/ncomms1663 22314359

[ecy70043-bib-0090] Ward, B. A. , and M. J. Follows . 2016. “Marine Mixotrophy Increases Trophic Transfer Efficiency, Mean Organism Size, and Vertical Carbon Flux.” Proceedings of the National Academy of Sciences of the United States of America 113: 2958–2963. 26831076 10.1073/pnas.1517118113 PMC4801304

[ecy70043-bib-0091] Warton, D. I. , and F. K. Hui . 2011. “The Arcsine Is Asinine: The Analysis of Proportions in Ecology.” Ecology 92: 3–10. 21560670 10.1890/10-0340.1

[ecy70043-bib-0092] Westoby, M. , and I. J. Wright . 2006. “Land‐Plant Ecology on the Basis of Functional Traits.” Trends in Ecology & Evolution 21: 261–268. 16697912 10.1016/j.tree.2006.02.004

[ecy70043-bib-0093] Winberg, G. G. 1971. Symbols, Units and Conversion Factors in Studies of Freshwater Productivity. London: The University of Chicago Press.

[ecy70043-bib-0094] Wolf, R. , and J. Heuschele . 2018. “Water Browning Influences the Behavioral Effects of Ultraviolet Radiation on Zooplankton.” Frontiers in Ecology and Evolution 6: 1–8.

[ecy70043-bib-0095] Wood, S. N. 2017. Generalized Additive Models: An Introduction with R, Second ed. New York, NY: Chapman and Hall/CRC.

[ecy70043-bib-0096] Yang, Z. , and F. Kong . 2012. “Formation of Large Colonies: A Defense Mechanism of *Microcystis aeruginosa* under Continuous Grazing Pressure by Flagellate *Ochromonas* sp.” Journal of Limnology 71(5): e5.

