# Supplementary material for: Cell size explains shift in phytoplankton community structure following storm‐induced changes in light and nutrients
Source: Ecology. 2025 Mar 11;106(3):e70043. doi: 10.1002/ecy.70043 (PMC11894364; doi:10.1002/ecy.70043)
Supplement: Supplementary file 1 — Appendix S1. [file ECY-106-e70043-s002.pdf]

Ecology

Cell size explains shift in phytoplankton community structure following storm-induced changes in light and nutrients

Alexis L. N. Guislain, Jens C. Nejstgaard, Jan Köhler, Erik Sperfeld, Ute Mischke, Birger Skjelbred, Hans-Peter Grossart, Anne Lyche Solheim, Mark O. Gessner, Stella A. Berger

Appendix S1 - Environmental conditions

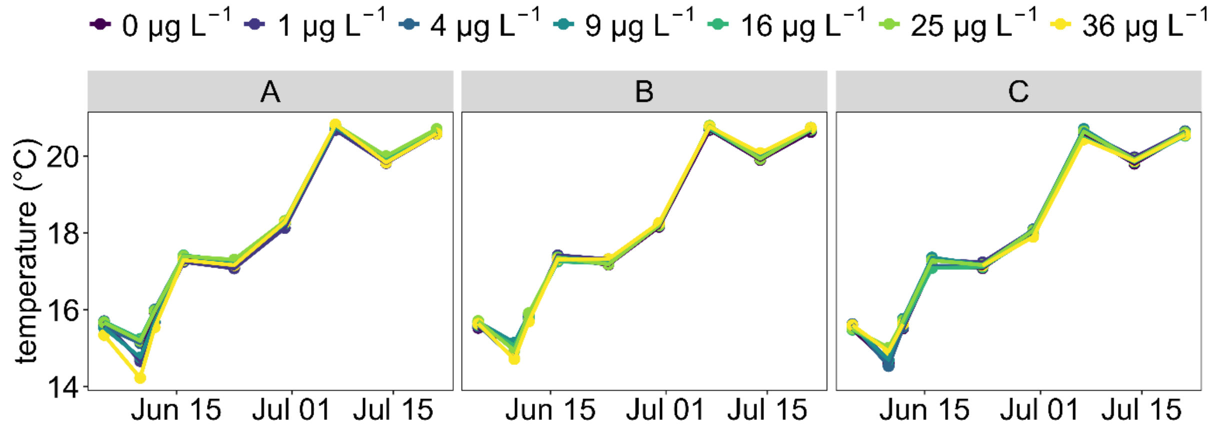

FIGURE S1 | Temporal dynamics of temperature in the epilimnion in enclosures receiving no (A: 0 mg HF L^{-1}), intermediate (B: 5 mg HF L^{-1}) or high (C: 10 mg HF L^{-1}) levels of cDOM. The initial experimental P enrichment (µg L^{-1}) is represented by the color gradient from dark blue to yellow.

TABLE S1 | Statistics of the LME model explaining the variation in temperature as a function of time and browning level, including standard errors (SE) and bootstrapped 95% confidence intervals (999 simulations).

Factor	Estimate	SE	95% CI	P-value
(Intercept)	15.05	0.15	14.78 – 15.33	<0.001
day	0.13	$5.71\ 10^{-3}$	0.12 – 0.14	<0.001
browning [B]	$2.89\ 10^{-2}$	0.21	- 0.39 – 0.44	0.89
browning [C]	- 0.13	0.21	- 0.56 – 0.29	0.53
day : browning [B]	$-9.14\ 10^{-4}$	$8.09\ 10^{-3}$	- 0.02 – 0.01	0.91
day : browning [C]	$8.37\ 10^{-4}$	$8.08\ 10^{-3}$	- 0.02 – 0.02	0.92

TABLE S2 | Mean temperatures (°C) in the epilimnion of enclosures receiving no (A: 0 mg HF L^{-1}), intermediate (B: 5 mg HF L^{-1}) or high (C: 10 mg HF L^{-1}) levels of cDOM.

Date	A	B	C
05 June	15.6	15.6	15.6
10 June	14.9	15.0	14.8
12 June	15.8	15.8	15.7
16 June	17.3	17.3	17.2
23 June	17.2	17.3	17.2
30 June	18.2	18.2	18.0
07 July	20.8	20.7	20.6
14 July	19.9	20.0	19.9
21 July	20.6	20.7	20.6

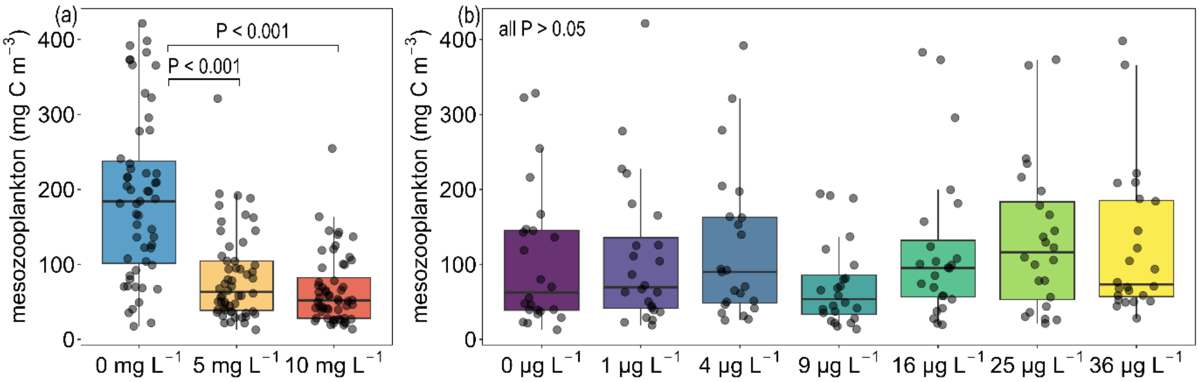

FIGURE S2 | Mesozooplankton biomass in enclosures receiving no (A: 0 mg HF L^{-1}), intermediate (B: 5 mg HF L^{-1}) or high (C: 10 mg HF L^{-1}) levels of cDOM (**a**), and at seven nutrient enrichment levels (addition of 0, 1, 4, 9, 16, 25 and 36 µg P L^{-1}) (**b**). P-values refer to the difference between each level of cDOM (**a**) or nutrient enrichment (**b**) and the intercept, i.e., the control enclosures with no HF or P addition, respectively.

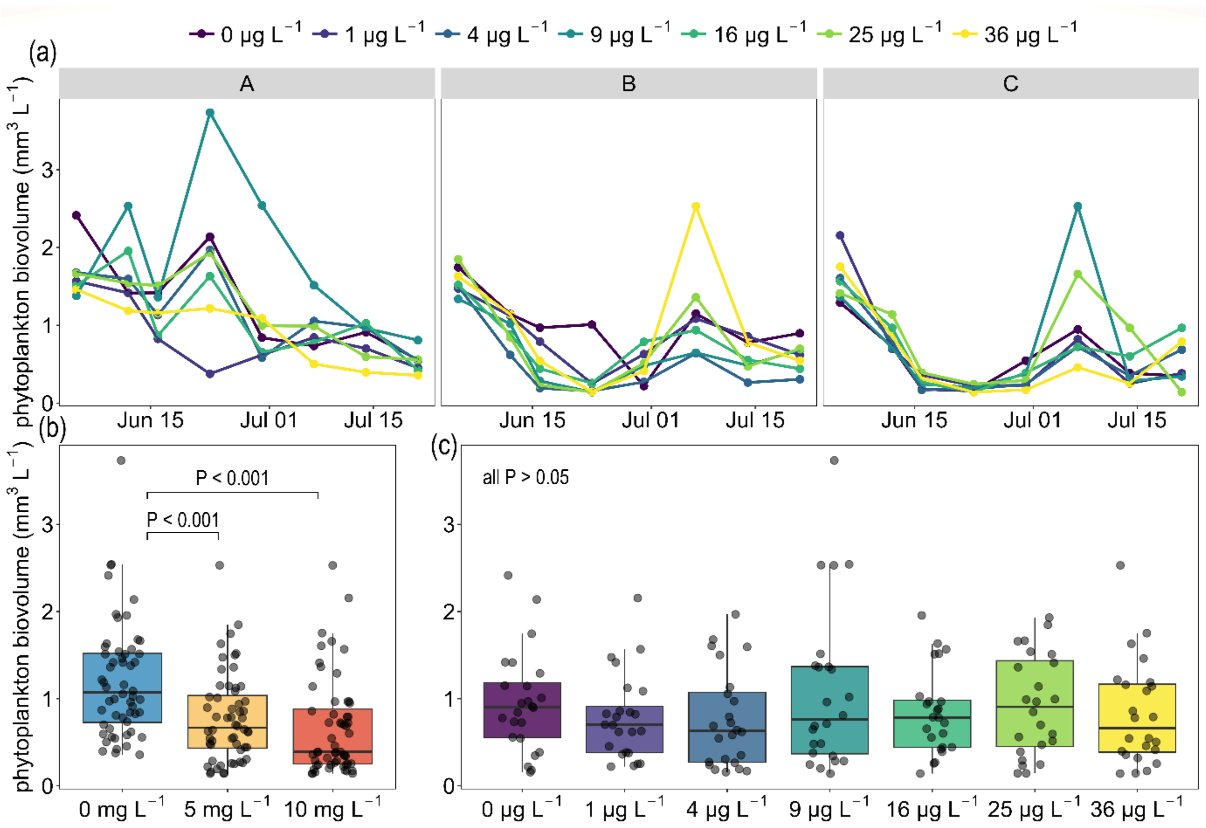

FIGURE S3 | Temporal dynamics of phytoplankton biovolume in enclosures receiving no (A: 0 mg HF L^{-1}), intermediate (B: 5 mg HF L^{-1}) or high (C: 10 mg HF L^{-1}) levels of cDOM (**a**). The initial experimental P enrichment (µg L^{-1}) is represented by the color gradient from dark blue to yellow. P-values refer to the difference between each level of cDOM (**b**) or nutrient enrichment (**b**) and the intercept, i.e., the control enclosures with no HF or P addition, respectively.
