# Supplementary material for: Cell size explains shift in phytoplankton community structure following storm‐induced changes in light and nutrients
Source: Ecology. 2025 Mar 11;106(3):e70043. doi: 10.1002/ecy.70043 (PMC11894364; doi:10.1002/ecy.70043)
Supplement: Supplementary file 2 — Appendix S2. [file ECY-106-e70043-s004.pdf]

Ecology

Cell size explains shift in phytoplankton community structure following storm-induced changes in light and nutrients

Alexis L. N. Guislain, Jens C. Nejstgaard, Jan Köhler, Erik Sperfeld, Ute Mischke, Birger Skjelbred, Hans-Peter Grossart, Anne Lyche Solheim, Mark O. Gessner, Stella A. Berger

Appendix S2 - Phytoplankton size distribution

We generated two types of distributions for each enclosure and sampling day. Firstly, we created frequency distributions of cell sizes of species represented by species numbers along the cell size gradient. Secondly, we calculated the biovolume-weighted cell size distributions of phytoplankton species, which are frequency distributions of cell sizes of species weighted by their respective biovolume (Downing et al. 2014). The biovolume-weighted size distribution provides insight into the distribution of biovolume across the cell size gradient within each enclosure. To estimate the probability density function of these distributions, we employed Kernel density estimates (Downing et al. 2014), with the bandwidth selected using the method proposed by Sheather and Jones (1991).

REFERENCES

Downing, A. S., S. Hajdu, O. Hjerne, S. A. Otto, T. Blenckner, U. Larsson, and M. Winder. 2014. Zooming in on size distribution patterns underlying species coexistence in Baltic Sea phytoplankton. *Ecology Letters* 17:1219–1227.

Sheather, S. J., and M. C. Jones. 1991. A Reliable Data-Based Bandwidth Selection Method for Kernel Density Estimation. *Journal of the Royal Statistical Society: Series B (Methodological)* 53:683–690.

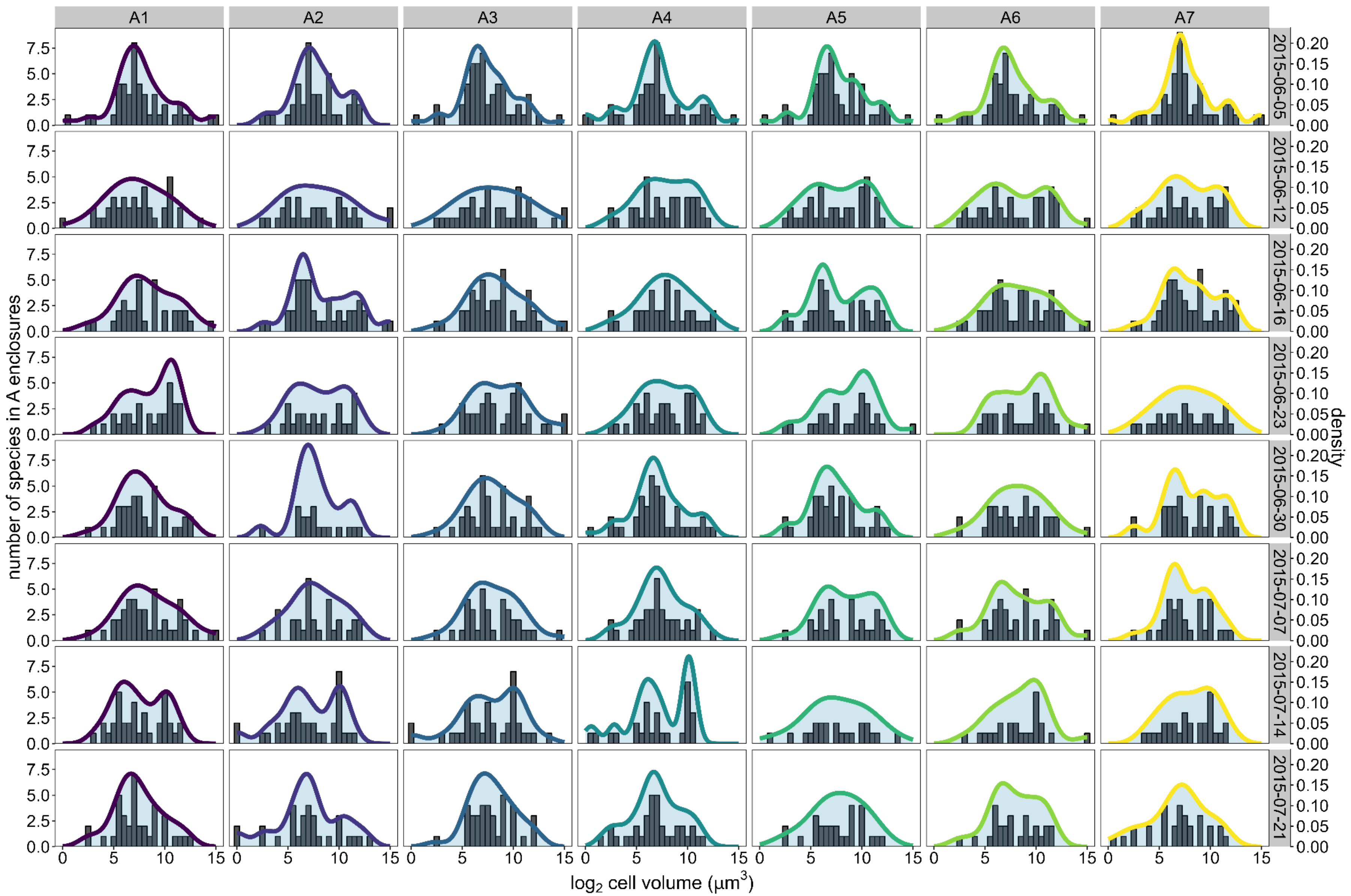
A1
A2
A3
A4
A5
A6
A7
2015-06-05
2015-06-12
2015-06-16
2015-06-23
2015-06-30
2015-07-07
2015-07-14
2015-07-21
number of species in A enclosures
density
log2 cell volume (μm3)
0.0
2.5
5.0
7.5
0.00
0.05
0.10
0.15
0.20
0
5
10
15

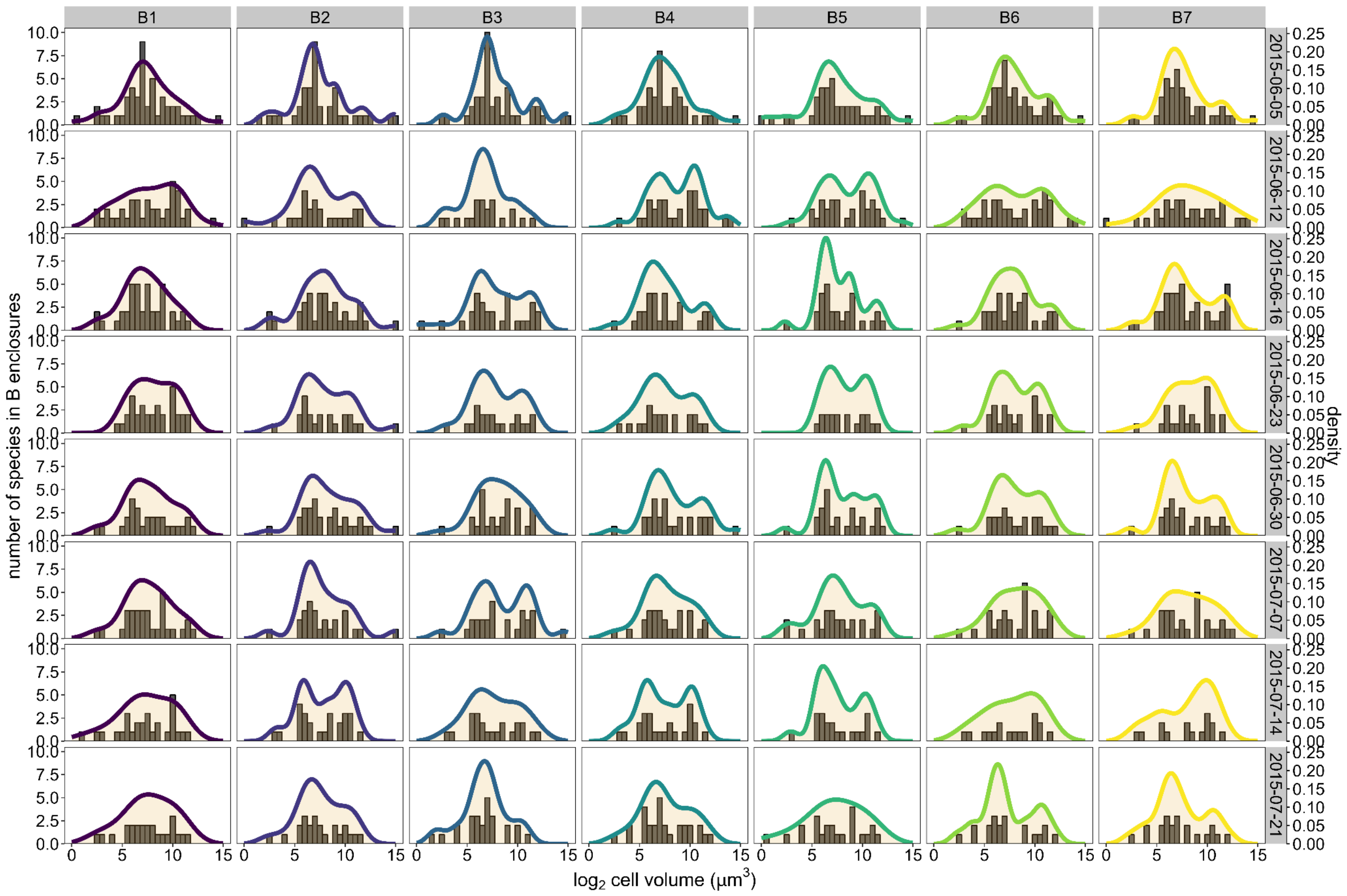

B1
B2
B3
B4
B5
B6
B7
2015-06-05
2015-06-12
2015-06-16
2015-06-23
2015-06-30
2015-07-07
2015-07-14
2015-07-21
number of species in B enclosures
density
$\log_2$ cell volume (μm^3)
0.0
2.5
5.0
7.5
10.0
0.00
0.05
0.10
0.15
0.20
0.25
0
5
10
15

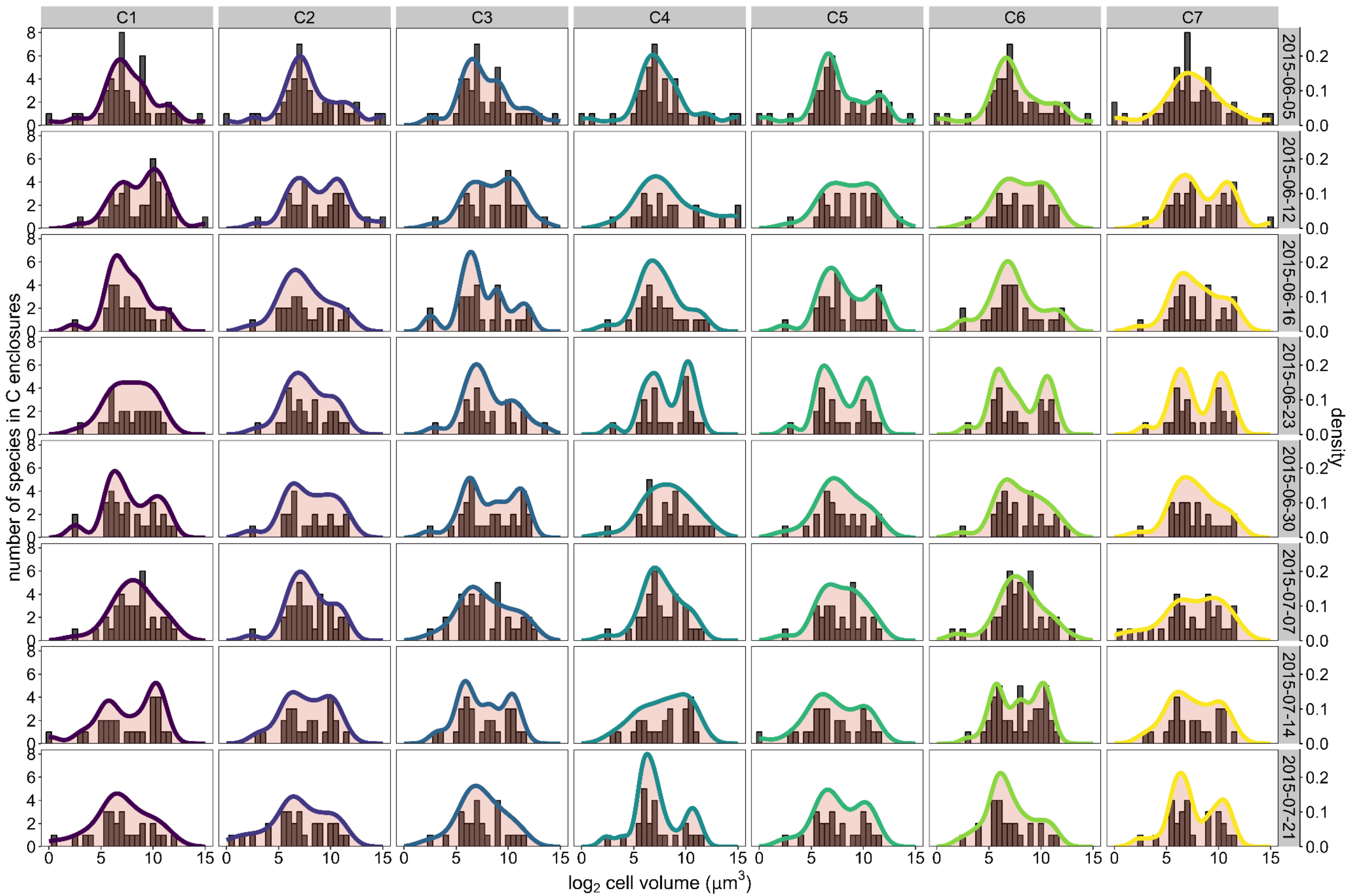
C1
C2
C3
C4
C5
C6
C7
2015-06-05
2015-06-12
2015-06-16
2015-06-23
2015-06-30
2015-07-07
2015-07-14
2015-07-21
number of species in C enclosures
density
log2 cell volume (μm3)

FIGURE S1 | Cell-size frequency distributions of phytoplankton species. Bars correspond to the primary y-axes and show species numbers. The curves corresponding to the secondary y-axes are Kernel density estimations of the probability density function. The 3-level browning gradient is represented by the filling color for enclosures A (blue, 0 mg HF L^{-1}), B (yellow, 5 mg HF L^{-1}) and C (red, 10 mg HF L^{-1}). The 7-level nutrient enrichment gradient (addition of 0, 1, 4, 9, 16, 25, and 36 µg P L^{-1}) is represented by the line color gradient and numbered from 1 (dark blue, no P addition) to 7 (yellow, 36 µg P L^{-1}).

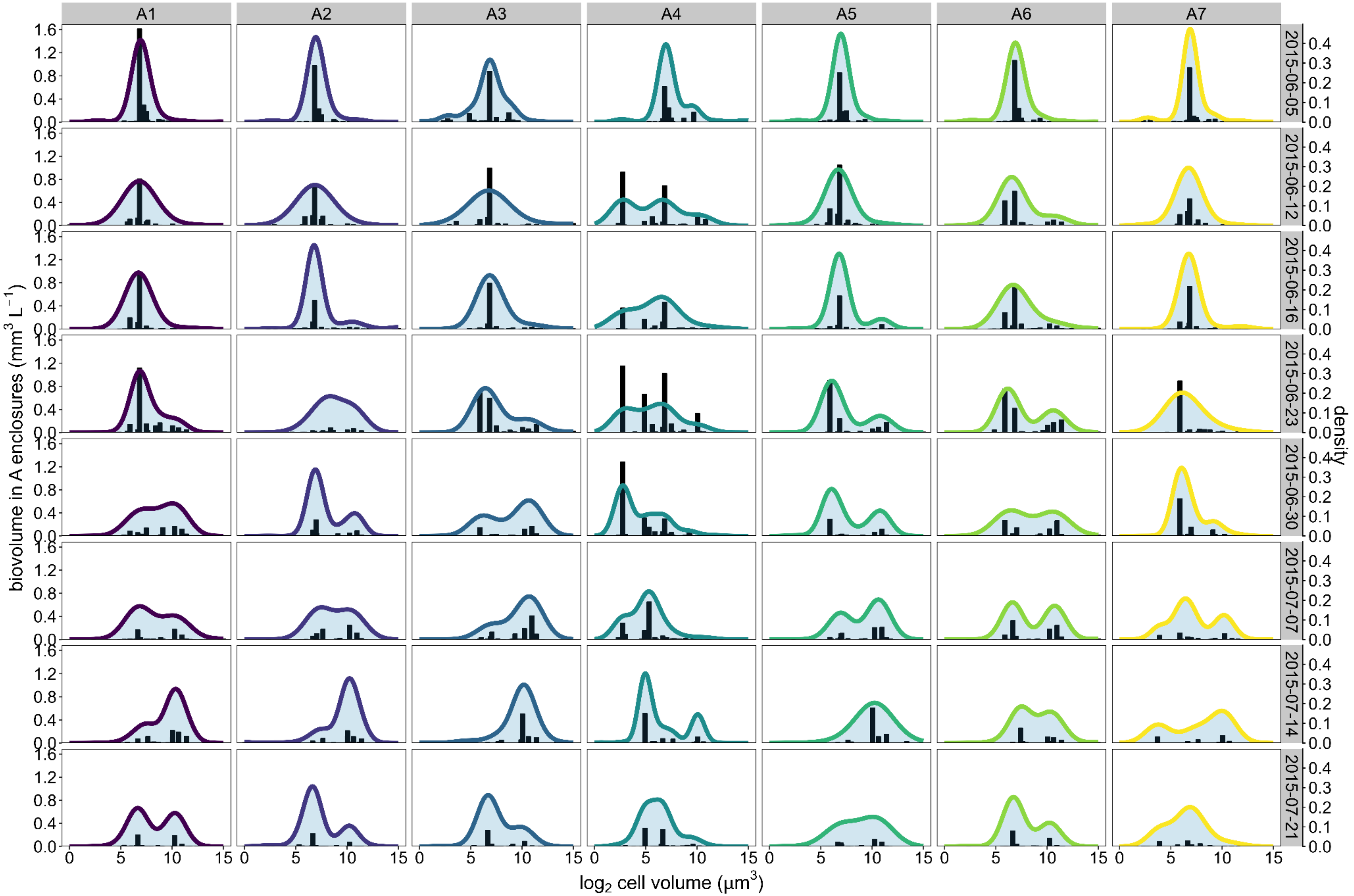

A1
A2
A3
A4
A5
A6
A7
2015-06-05
2015-06-12
2015-06-16
2015-06-23
2015-06-30
2015-07-07
2015-07-14
2015-07-21
biovolume in A enclosures (mm^3 L^{-1})
density
log$_2$ cell volume (μm^3)

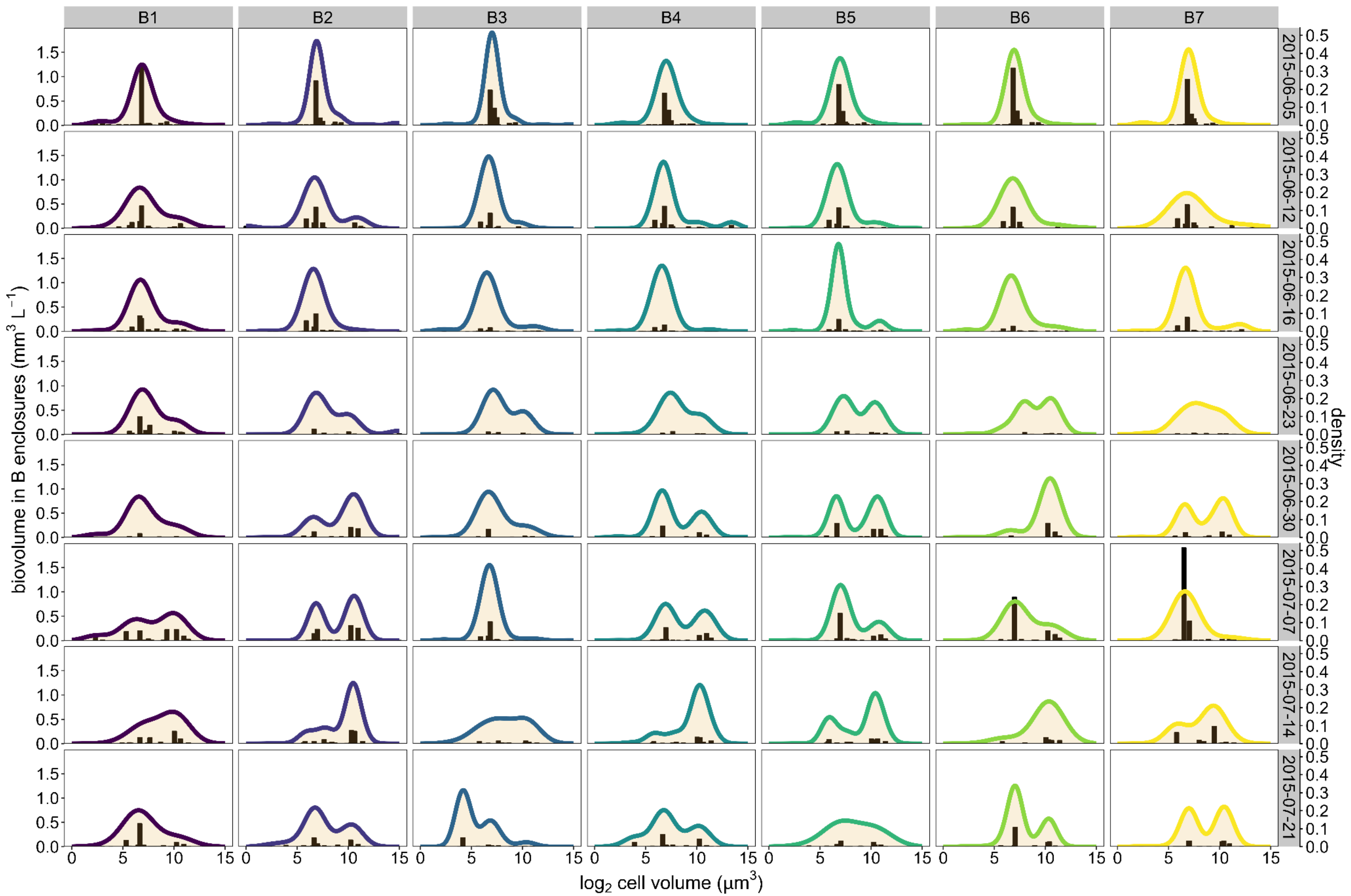
B1
B2
B3
B4
B5
B6
B7
2015-06-05
2015-06-12
2015-06-16
2015-06-23
2015-06-30
2015-07-07
2015-07-14
2015-07-21
biovolume in B enclosures (mm³ L⁻¹)
density
log₂ cell volume (µm³)

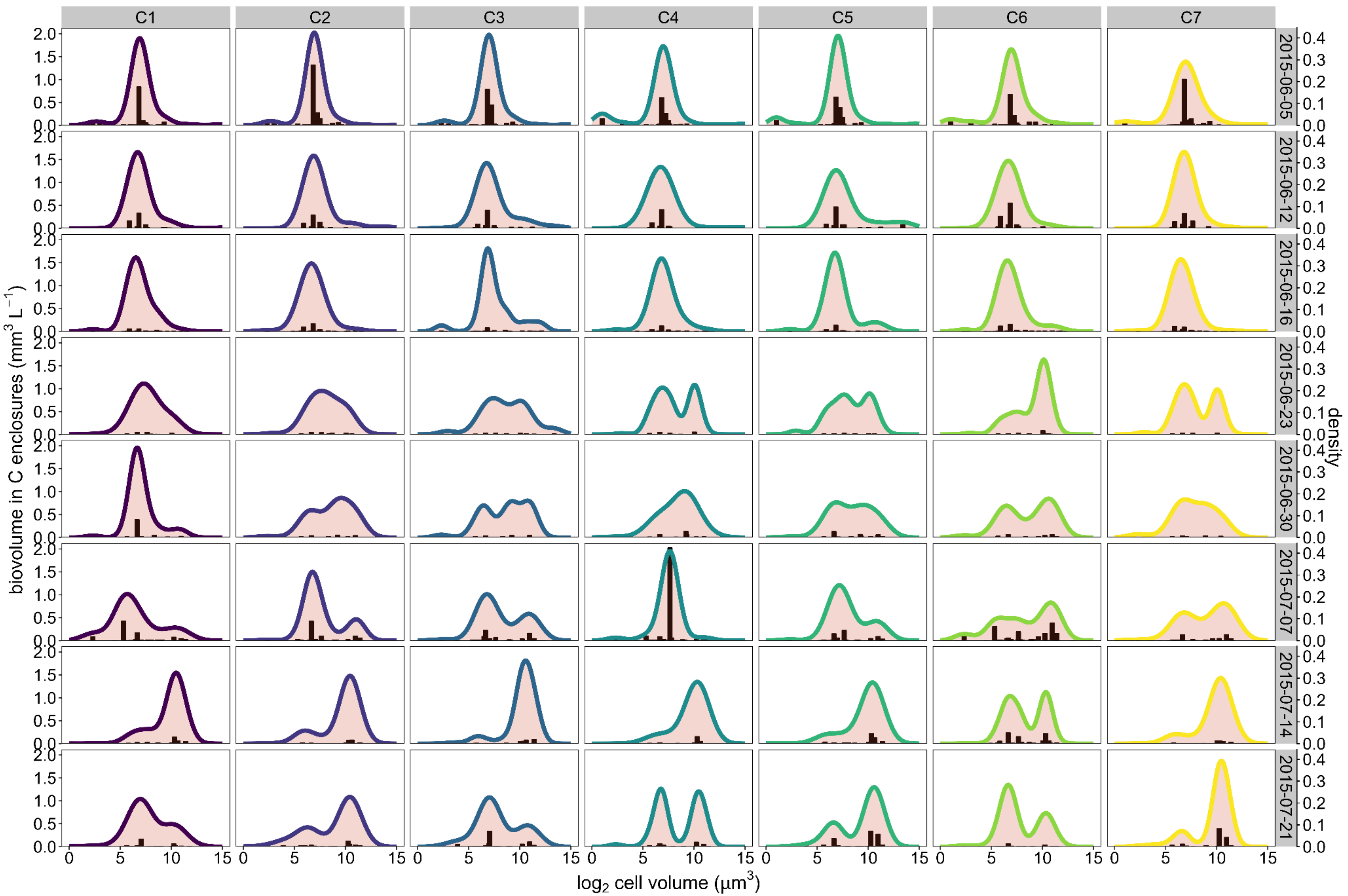

C1
C2
C3
C4
C5
C6
C7
2015-06-05
2015-06-12
2015-06-16
2015-06-23
2015-06-30
2015-07-07
2015-07-14
2015-07-21
biovolume in C enclosures (mm^3 L^{-1})
density
$\log_2$ cell volume (μm^3)
0.0
0.5
1.0
1.5
2.0
0.1
0.2
0.3
0.4
0
5
10
15

FIGURE S2 | Biovolume-weighted cell size distributions of phytoplankton in all enclosures throughout the experiment. Bars correspond to the primary y-axes and show absolute biovolume (mm^3 L^{-1}). The curves corresponding to the secondary y-axes are Kernel density estimations of the probability density function. The 3-level browning gradient is represented by the filling color gradient for enclosures A (blue, 0 mg HF L^{-1}), B (yellow, 5 mg HF L^{-1}) and C (red, 10 mg HF L^{-1}). The 7-level nutrient enrichment gradient (addition of 0, 1, 4, 9, 16, 25, and 36 µg P L^{-1}) is represented by the line color gradient and numbered from 1 (dark blue, no P addition) to 7 (yellow, 36 µg P L^{-1}).

TABLE S1 | Results of Generalized Additive Models (GAMs) of the logit-transformed relative biovolume of phytoplankton species in different size categories. The estimates, standard errors (SE), t-value and P-values of the parametric coefficient intercept are presented (**a**). The effective degrees of freedom (edf), the reference degrees of freedom (Ref.df), the F-values and the P-values of the smooth terms are presented (**b**).

(a)

Model	Time experiment	Browning level	Parametric coefficient	Estimate	SE	t-value	P-value
gam1A	start	A	(Intercept)	-4.2	0.1	-33.1	<0.001
gam1B	start	B	(Intercept)	-4.1	0.1	-48.4	<0.001
gam1C	start	C	(Intercept)	-3.8	0.1	-68.0	<0.001
gam2A	end	A	(Intercept)	-3.8	0.2	-18.8	<0.001
gam2B	end	B	(Intercept)	-3.6	0.2	-17.2	<0.001
gam2C	end	C	(Intercept)	-3.4	0.1	-24.6	<0.001

(b)

Model	Time experiment	Browning level	Smooth terms	edf	Ref.df	F	P-value
gam1A	start	A	s(size_category)	10.9	11.8	36.6	<0.001
gam1A	start	A	s(phosphate)	2.8	6.0	0.8	0.10
gam1B	start	B	s(size_category)	11.5	11.9	77.0	<0.001
gam1B	start	B	s(phosphate)	2.6	6.0	0.8	0.12
gam1C	start	C	s(size_category)	11.8	12.0	142.7	<0.001
gam1C	start	C	s(phosphate)	2.3	6.0	0.6	0.16
gam2A	end	A	s(size_category)	6.3	7.6	9.4	<0.001
gam2A	end	A	s(phosphate)	$3.5\ 10^{-4}$	6.0	0	0.77
gam2B	end	B	s(size_category)	6.7	8.0	9.4	<0.001
gam2B	end	B	s(phosphate)	$1.4\ 10^{-3}$	6.0	0	0.44
gam2C	end	C	s(size_category)	9.5	9.9	15.8	<0.001
gam2C	end	C	s(phosphate)	$7.5\ 10^{-5}$	6.0	0	1
