# Supplementary material for: Cell size explains shift in phytoplankton community structure following storm‐induced changes in light and nutrients
Source: Ecology. 2025 Mar 11;106(3):e70043. doi: 10.1002/ecy.70043 (PMC11894364; doi:10.1002/ecy.70043)
Supplement: Supplementary file 3 — Appendix S3. [file ECY-106-e70043-s001.pdf]

Ecology

Cell size explains shift in phytoplankton community structure following storm-induced changes in light and nutrients

Alexis L. N. Guislain, Jens C. Nejstgaard, Jan Köhler, Erik Sperfeld, Ute Mischke, Birger Skjelbred, Hans-Peter Grossart, Anne Lyche Solheim, Mark O. Gessner, Stella A. Berger

Appendix S3 – Species number

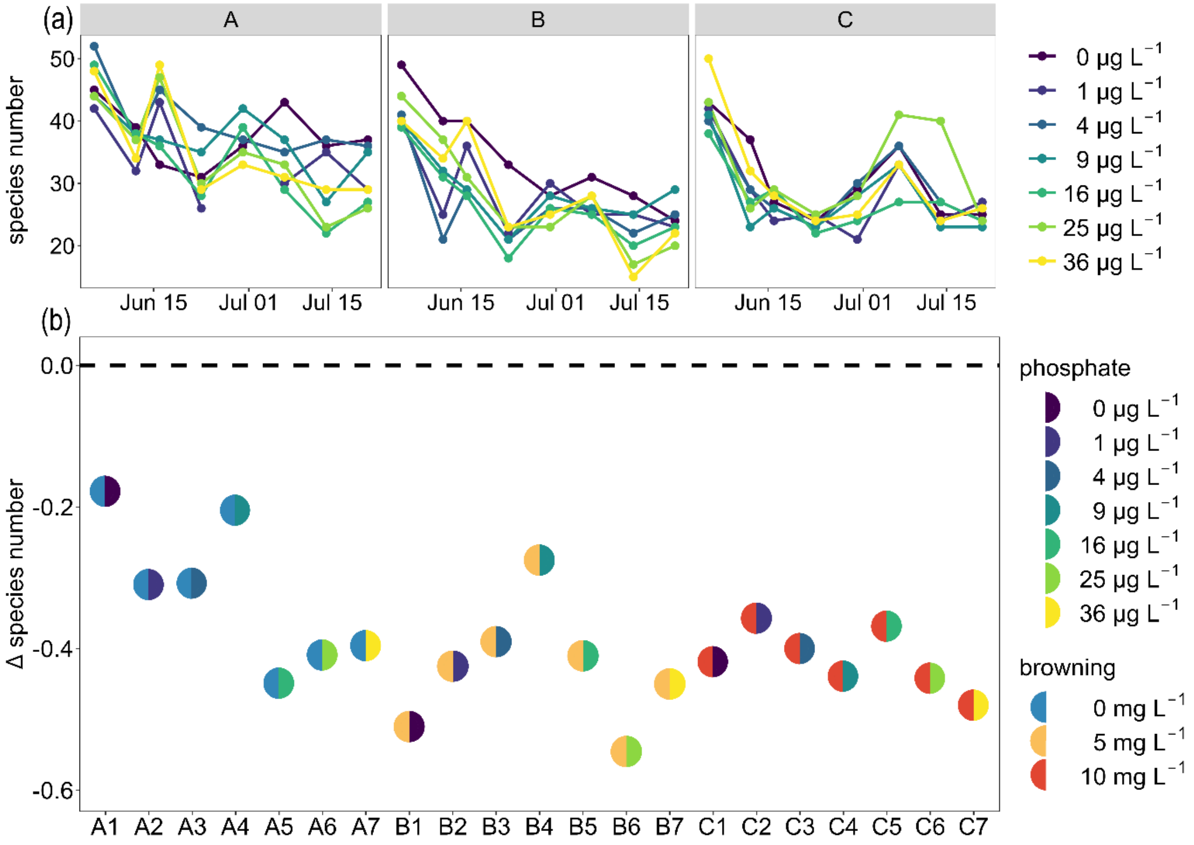

FIGURE S1 | Temporal dynamics of phytoplankton species numbers in enclosures receiving no (A: 0 mg HF L^{-1}), intermediate (B: 5 mg HF L^{-1}) or high (C: 10 mg HF L^{-1}) levels of cDOM (**a**) and percentage of variation in phytoplankton species numbers in all enclosures between 5 June and 21 July (**b**). The 7-level nutrient enrichment gradient (addition of 0, 1, 4, 9, 16, 25, and 36 µg P L^{-1}) is numbered from 1 (dark blue, no P addition) to 7 (yellow, 36 µg P L^{-1}).

Phytoplankton species numbers declined in all enclosures throughout the experiment (**Figure S1a**). It is noteworthy that species numbers may have tended to rebound towards the end of the experiment in the C enclosures. All enclosures combined, an average of 39 ± 9% (±SD) of the initial richness was lost between the first and last day of the experiment (**Figure S1b**), with losses in individual enclosures ranging between 18 and 54%. Elapsed time was thus included in our initial linear mixed effects model which hypothesized species richness to

respond to the effects of PAR, SRP and mesozooplankton biomass. Elapsed time had a significant effect on species numbers as demonstrated by the LRT between the model that included the day of experiment and the model that did not (LRT, $X^2 = 71.0$, $P < 0.001$). The effect of mesozooplankton biomass was not significant (LRT, $X^2 = 0.96$, $P = 0.33$) and, zooplankton biomass was thus dropped from the model. Results were supported by the lowest AIC. Although temperature was positively correlated with time, replacing elapsed time with temperature would alter the model (delta AIC = 25). Thus, the species number model was:

$$number_i^j = \beta_0^j + \beta_{day} day_i + \beta_{PAR} PAR_i + \beta_{SRP} SRP_i + \beta_{PAR*SRP} PAR_i * SRP_i + \varepsilon_i^j \quad (1),$$

where $number_i^j$ is the phytoplankton species number in the enclosure j for observation i. Term β_0^j is the enclosure-specific intercept for enclosure j, β_{day} the coefficient for day of experiment, day_i the standardized day of experiment for observation i, β_{PAR} the coefficient for PAR, PAR_i the standardized mean PAR in the epilimnion during daytime for observation i, β_{SRP} the coefficient for SRP, SRP_i the standardized SRP for observation i, $\beta_{PAR*SRP}$ the coefficient for PAR*SRP and ε_i^j is the residual species number. The enclosure-specific intercept for enclosure j, β_0^j, is expressed as $\beta_0^j = \beta_0 + u_0^j$. Term u_0^j is the random effect for enclosure j, which is normally distributed with mean zero and variance $\sigma_0{}^2$ and estimates unexplained interspecific variation in the intercept. Term β_0 is the fixed intercept. Model diagnostics showed satisfactory agreement with the assumptions of linearity, homogeneity of variance, normality of residuals and normality of random effects.

Species number increased significantly with increasing light availability (PAR, $P < 0.001$; **Figure S2, Table S1**). In contrast, species number declined throughout the experiment (DAY, $P < 0.001$). There was no significant effect of SRP on the species numbers ($P = 0.79$) and the interaction between PAR and SRP was also found to be non-significant ($P = 0.06$). The lack of response of species number to SRP concentrations was further demonstrated in

the A enclosures, by computing the LRT comparing the model with elapsed time as sole explaining variable of richness and the model with added effects of time and SRP (LRT, $X^2 < 0.1$, $P = 0.99$).

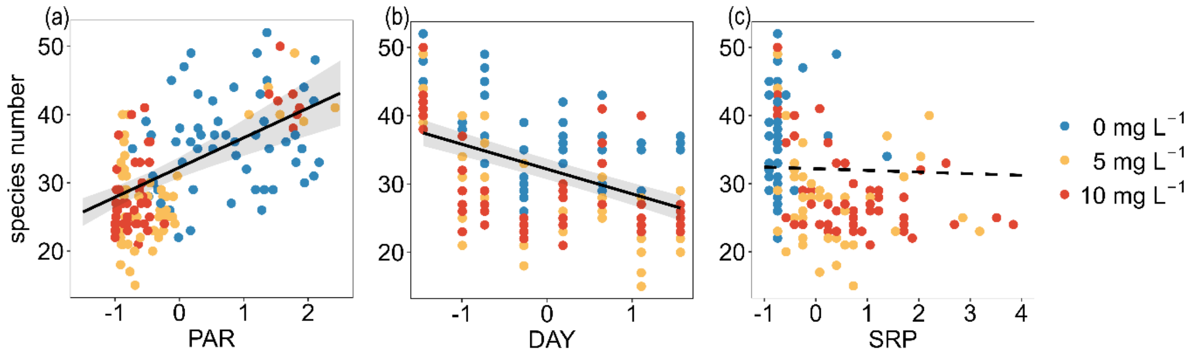

FIGURE S2 | The relationship between phytoplankton species number and standardized PAR (**a**), day of experiment (**b**) and SRP (**c**). The enclosures A (0 mg HF L^{-1}), B (5 mg HF L^{-1}) and C (10 mg HF L^{-1}) are represented by the color code. The significant effects ($P < 0.001$) in the model are represented with solid lines and 95% confidence intervals.

TABLE S1 | Statistics of the LME model (see equation 1 in Appendix S3) explaining the variation in phytoplankton species number as a function PAR, SRP and day of experiment, including standard errors (SE) and bootstrapped 95% confidence intervals (999 simulations).

Factor	Estimate	SE	95% CI	P-value
β_0	32.25	0.76	30.77; 33.68	< 0.001
β_{day}	-3.63	0.38	-4.34; -2.90	< 0.001
β_{PAR}	4.34	0.78	2.71; 5.87	< 0.001
β_{SRP}	-0.22	0.81	-1.83; 1.45	0.79
$\beta_{PAR*SRP}$	1.72	0.91	-0.17; 3.53	0.06
