# Supplementary material for: Cell size explains shift in phytoplankton community structure following storm‐induced changes in light and nutrients
Source: Ecology. 2025 Mar 11;106(3):e70043. doi: 10.1002/ecy.70043 (PMC11894364; doi:10.1002/ecy.70043)
Supplement: Supplementary file 4 — Appendix S4. [file ECY-106-e70043-s003.pdf]

Ecology

Cell size explains shift in phytoplankton community structure following storm-induced changes in light and nutrients

Alexis L. N. Guislain, Jens C. Nejstgaard, Jan Köhler, Erik Sperfeld, Ute Mischke, Birger Skjelbred, Hans-Peter Grossart, Anne Lyche Solheim, Mark O. Gessner, Stella A. Berger

Appendix S4 – The biovolume models

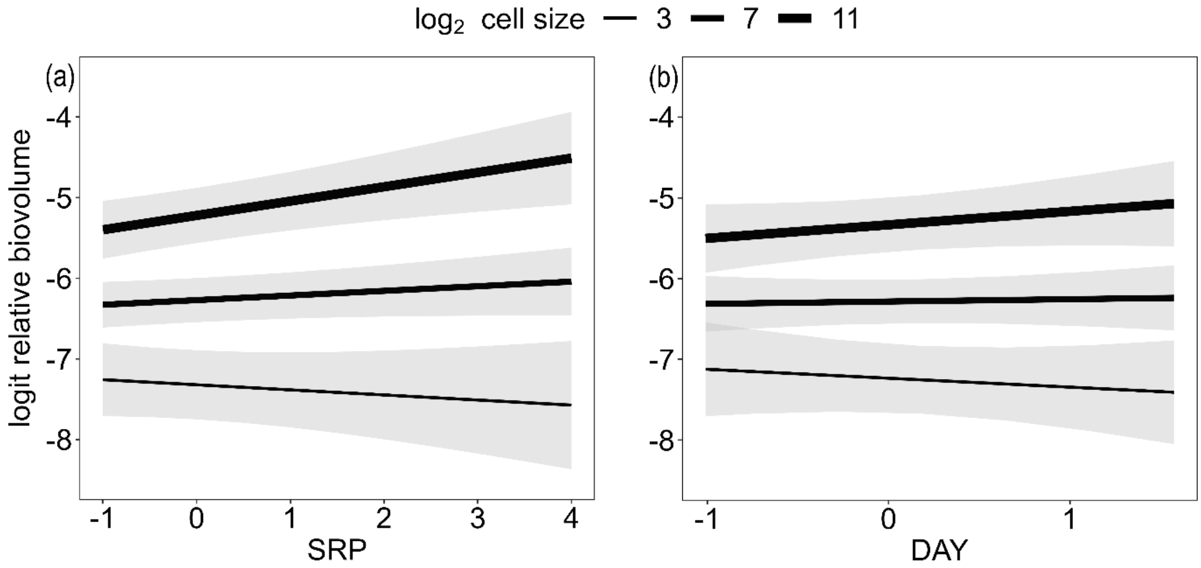

FIGURE S1 | Predicted relationships between cell size and relative phytoplankton biovolume along gradients of standardized SRP (**a**) and elapsed time (**b**). Bands represent 95% confidence intervals. The three lines per plot indicate species with cell sizes of 3, 7 and 11 $\log_2 \mu m^3$, corresponding to 8, 128 and 2048 μm^3.
